# A transgenerational role of the germline nuclear RNAi pathway in repressing heat stress-induced transcriptional activation in *C. elegans*

**DOI:** 10.1186/s13072-016-0052-x

**Published:** 2016-01-15

**Authors:** Julie Zhouli Ni, Natallia Kalinava, Esteban Chen, Alex Huang, Thi Trinh, Sam Guoping Gu

**Affiliations:** Department of Molecular Biology and Biochemistry, Rutgers the State University of New Jersey, Piscataway, NJ 08854 USA; Nelson Labs A125, 604 Allison Road, Piscataway, NJ 08854 USA

**Keywords:** Nuclear RNAi, Heat stress, Nuclear Argonaute protein HRDE-1/WAGO-9, LTR retrotransposon, Retrotransposon silencing, Mortal germline phenotype (*Mrt*), Transcriptional silencing, Heterochromatin, Germline, ChIP-seq, RNA-seq

## Abstract

**Background:**

Environmental stress-induced transgenerational epigenetic effects have been observed in various model organisms and human. The capacity and mechanism of such phenomena are poorly understood. In *C. elegans,* siRNA mediates transgenerational gene silencing through the germline nuclear RNAi pathway. This pathway is also required to maintain the germline immortality when *C. elegans* is under heat stress. However, the underlying molecular mechanism is unknown. In this study, we investigated the impact of heat stress on chromatin, transcription, and siRNAs at the whole-genome level, and whether any of the heat-induced effects is transgenerationally heritable in either the wild-type or the germline nuclear RNAi mutant animals.

**Results:**

We performed 12-generation temperature-shift experiments using the wild-type *C. elegans* and a mutant strain that lacks the germline-specific nuclear Argonaute protein HRDE-1/WAGO-9. By examining the mRNA, small RNA, RNA polymerase II, and H3K9 trimethylation profiles at the whole-genome level, we revealed an epigenetic role of HRDE-1 in repressing heat stress-induced transcriptional activation of over 280 genes. Many of these genes are in or near LTR (long-terminal repeat) retrotransposons. Strikingly, for some of these genes, the heat stress-induced transcriptional activation in the *hrde*-*1* mutant intensifies in the late generations under the heat stress and is heritable for at least two generations after the mutant animals are shifted back to lower temperature. *hrde*-*1* mutation also leads to siRNA expression changes of many genes. This effect on siRNA is dependent on both the temperature and generation.

**Conclusions:**

Our study demonstrated that a large number of the endogenous targets of the germline nuclear RNAi pathway in *C. elegans* are sensitive to heat-induced transcriptional activation. This effect at certain genomic loci including LTR retrotransposons is transgenerational. Germline nuclear RNAi antagonizes this temperature effect at the transcriptional level and therefore may play a key role in heat stress response in *C. elegans*.

**Electronic supplementary material:**

The online version of this article (doi:10.1186/s13072-016-0052-x) contains supplementary material, which is available to authorized users.

## Background

RNA interference (RNAi) includes a diverse set of small RNA-guided gene silencing phenomena. It was initially discovered as a biochemical pathway in which double-stranded RNA (dsRNA) leads to degradation of target mRNA in a highly sequence-specific manner [1, 2]. In addition to the post-transcriptional silencing mechanism in the cytoplasm (referred to as classical RNAi), RNAi also occurs in the nucleus and represses gene expression at the chromatin level (referred to as nuclear RNAi) (reviewed in [3–7]). In plants and *Schizosaccharomyces pombe*, small interfering RNA (siRNA)-directed chromatin modifications (DNA methylation for plants and H3K9 methylation [H3K9me] for *S. pombe*) lead to transcriptional repression.

Most of our knowledge of nuclear RNAi in animals came from recent studies using *C. elegans*. Similar to plants and *S. pombe*, secondary siRNAs in *C. elegans*, either triggered by exogenous dsRNA or endogenous silencing RNA (e.g., piRNAs), can guide nuclear Argonaute proteins, together with other protein factors, to target genes for heterochromatin formation, marked by H3K9 trimethylation (H3K9me3), and transcriptional silencing [8–12]. Despite these similarities, several features of nuclear RNAi in *C. elegans* make this pathway a unique model system to explore novel mechanisms of RNA-mediated chromatin silencing and its roles in developmental regulation. For example, nuclear RNAi occurs in both somatic and germ cells in *C. elegans,* each using a different AGO protein (NRDE-3 in soma and HRDE-1/WAGO-9 in germ cells) [9, 10, 13, 14]. HRDE-1/WAGO-9 is one of several worm-specific AGO proteins [15] and is essential for the heritable H3K9me3 response and heritable gene silencing triggered by exogenous dsRNA, piRNA, or transgene [10, 12–14]. These findings have established nuclear RNAi in *C. elegans* as an important model system to study RNA-mediated chromatin regulation and transgenerational epigenetic inheritance. (The name HRDE-1 is used in the rest of this article to reflect the *h*eritable *R*NAi-*de*ficient phenotype of the mutant.)

Despite recent progress in this area, the native function of the nuclear RNAi pathway in *C. elegans* is largely unknown. Based on the profile of HRDE-1-bound endogenous siRNAs [10, 14], a large number of protein-coding genes can potentially be targeted by the germline nuclear RNAi pathway. However, our previous study showed that only a small fraction of these candidate target genes become transcriptionally derepressed or exhibit loss of H3K9me3 in the germline nuclear RNAi mutants [16], suggesting that the expression of these genes, and therefore the requirement of germline nuclear RNAi in gene silencing, may be conditional.

Germline nuclear RNAi mutant animals are viable but more sensitive to heat stress than the wild-type (WT) animals. Intriguingly, it takes several generations of heat stress for the mutant population to become completely sterile [10, 13]. Prior to the generation that reaches complete sterility, the mutant population exhibits a progressive decline in fertility from one generation to the next. This so-called mortal germline (*Mrt*) phenotype indicates that the germline nuclear RNAi pathway plays a transgenerational role in maintaining the germline fitness under heat stress. The underlying molecular mechanism, however, remains elusive.

In our previous study [16], a permissive temperature (19 °C) was used so we could obtain a large amount of germline nuclear RNAi mutant worms. Given that the *Mrt* phenotype occurs at 23–25 °C, we decided to characterize the impact of heat stress on chromatin, transcription, and small RNA at the whole-genome level in the germline nuclear RNAi mutants, and, more importantly, determine whether any of the heat-induced effects is transgenerational heritable.

In this article, we reported that the expression of a subset of the germline nuclear RNAi target genes is temperature sensitive. Germline nuclear RNAi is dispensable for the silencing states of these genes at low temperature, but is required when animals are under even a mild heat stress. In the nuclear RNAi-defective mutant (*hrde*-*1*), a mild heat stress causes transcriptional activation at these genomic regions and this effect at many genomic loci is transgenerationally heritable.

## Results

### The design of multigenerational temperature-shift experiment

**Fig. 1:**
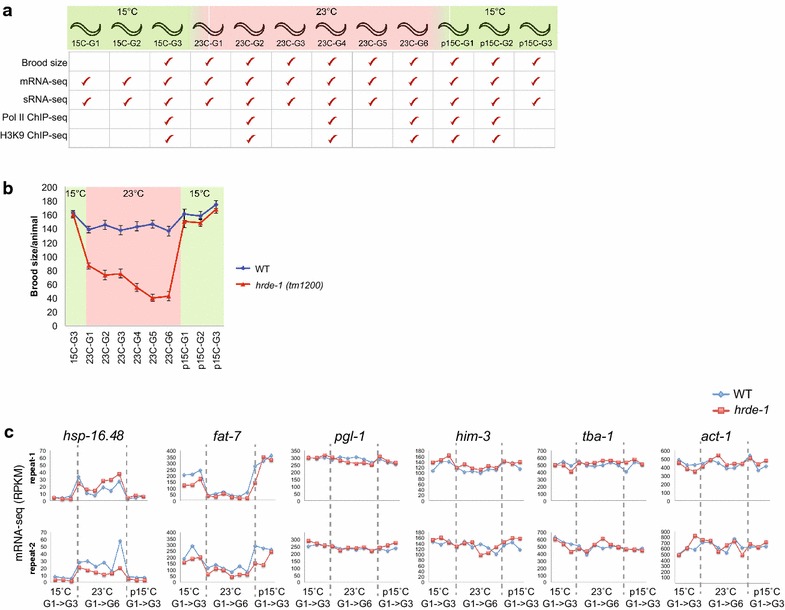
Multigenerational temperature-shift experiments using the N2 (WT) and *hrde*-*1*(*tm1200*) mutant strains. **a** Overall design. **b** Average brood size as a function of generation with temperature shift. **c** mRNA expression profiles of known temperature-sensitive genes and temperature-insensitive genes

To investigate the role of germline nuclear RNAi in transgenerational gene regulation and heat stress response, we performed a 12-generation temperature-shift experiment using the wild-type (N2) and *hrde*-*1*(*tm1200*) mutant strains. Two biological repeats were performed. In each repeat, developmentally synchronized animals were first cultured at 15 °C for three generations (referred to as 15C-G1, 15C-G2, and 15C-G3, “G” for **G**eneration) and then at 23 °C for six generations (23C-G1 to 23C-G6), followed by another three generations at 15 °C (p15C-G1, p15C-G2, and p15C-G3, “p” for ***p***ost-heat stress) (Fig. 1a). Young adult animals at each generation were collected for various analyses (Fig. 1a).

### The *Mrt* phenotype is reversible for the *hrde*-*1* mutant

As expected for the *Mrt* phenotype, the brood size of the *hrde*-*1* mutant population progressively decreased at 23 °C. (This limited the 23 °C-generation number for our assay.) Intriguingly, when shifted back to 15 °C, the population size quickly recovered. To verify this observation, we performed a brood-size analysis with individual worms using a similar temperature-shift scheme (Fig. 1a). We found that the average brood size of 23C-G6 decreased to approximately 25 % of the starting level at 15 °C, but the ones of post-heat stress generations (p15C-G1, G2, and G3) recovered to the pre-heat stress level (Fig. 1b). This result suggests that the molecular defect that causes the *Mrt* phenotype in the *hrde*-*1* mutant is transgenerationally cumulative *and* reversible. In addition, the *hrde*-*1* mutant strain has been cultured in our lab for 3 years (at both small and large scales) and we did not observe morphological phenotypes such as *Dpy* or *Unc*. Taken together, these results disfavor the idea that the *Mrt* phenotype of the nuclear RNAi-defective mutants is caused by DNA mutations.

## Germline nuclear AGO protein HRDE-1 prevents heat activation in mRNA expression for a subset of its native target genes

For our 12-generation mRNA-seq profiling analysis, we first verified that *hsp*-*16.48*, a heat shock protein gene [17], and *fat*-*7*, a cold-induced gene [18], exhibited the expected temperature-dependent mRNA expression profiles in both the WT and *hrde*-*1* mutant animals and in both biological repeats (Fig. 1c). As negative controls, many “house-keeping” genes and germline-specific genes showed relative stable expression under different temperatures in both *hrde*-*1* mutant and WT (some examples shown in Fig. 1c). These results confirm the temperature shift in our experiment.

**Fig. 2:**
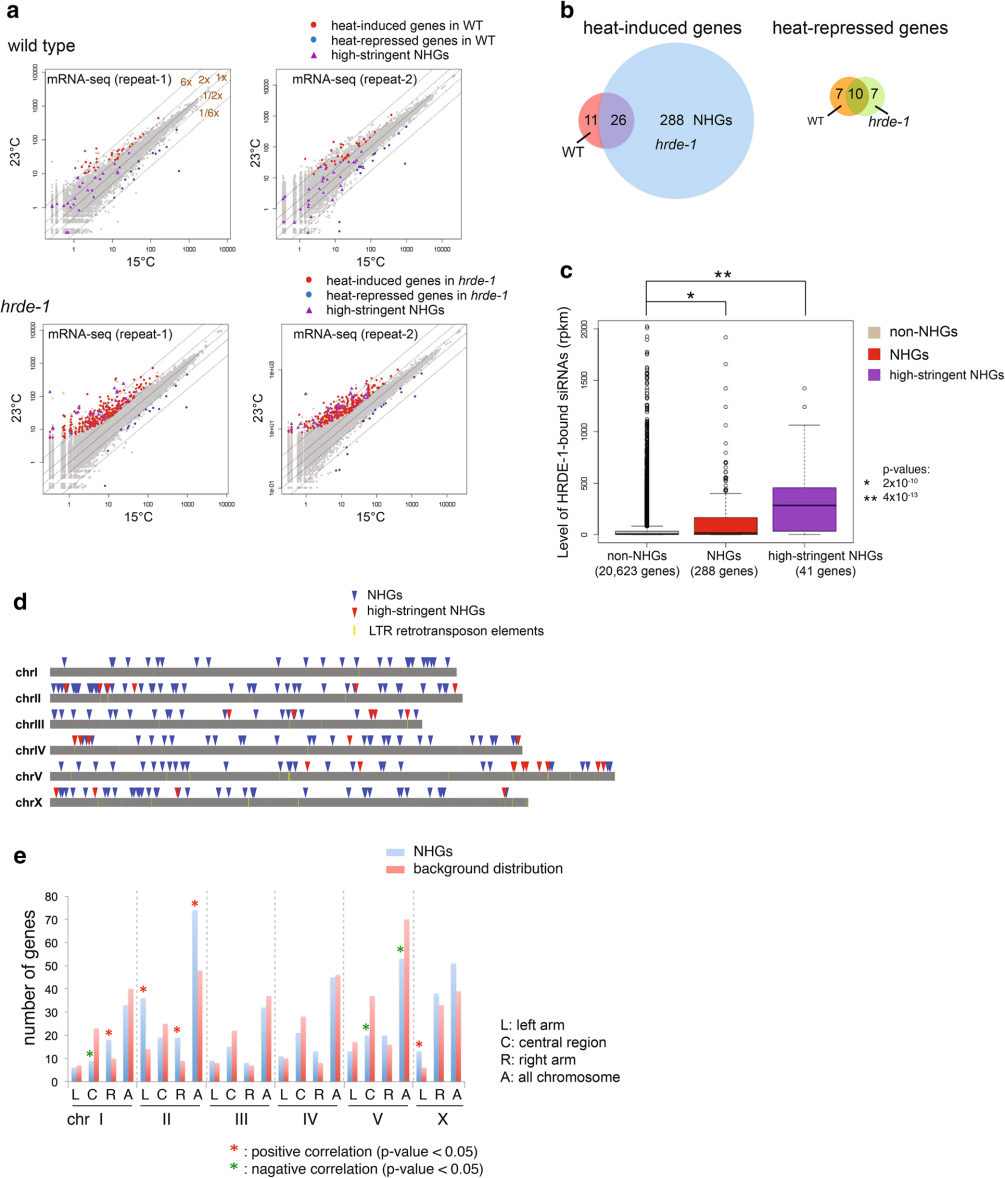
Germline nuclear AGO protein HRDE-1 prevents heat activation in mRNA expression for a subset of its native target genes. **a** Scatter plots comparing the 15 and 23 °C transcriptomes for WT and *hrde*-*1* mutant. Each dot corresponds to a protein-coding gene. Heat-induced genes and heat-repressed genes in WT or *hrde*-*1* mutant, as well as high-stringent NHGs (nuclear RNAi-repressed heat-inducible genes) are labeled as indicated. **b** Venn diagram analysis of temperature-sensitive genes in WT and *hrde*-*1* mutant. **c** Box plot analysis of HRDE-1-bound siRNAs for non-NHGs, NHGs, and high-stringent NHGs. 3XFLAG-HRDE-1 coIP siRNA sequencing data from [11] were used. Wilcoxon rank-sum test was used to calculate the *p* values. **d** Genomic distributions of NHGs, high-stringent NHGs, and LTR retrotransposons. **e** Number of NHGs located in the arms (1/4 of the chromosome length on each end) and central region (1/2 of chromosome length in the center) for each of the five autosomes. X chromosome was divided into two parts at the 1/6 of the length. *p* values were calculated by using the Chi-square test

To our knowledge, comparison between the 15 and 23 °C transcriptomes has not been reported for the wild-type *C. elegans*. To resolve this gap, we calculated the mean 15 °C mRNA-seq signal for each gene using the 15C-G1, 15C-G2, and 15C-G3 samples and the 23 °C one using the 23C-G2, 23C-G3, 23C-G4, 23C-G5, and 23-G6 samples (Fig. 2a). The 23C-G1 samples were excluded from this analysis because they experienced both temperatures, the 15 °C (from fertilization to egg prep) and 23 °C (from egg prep to young adult). By using a minimal twofold change (FDR ≤0.1) as the cutoff, we found 37 heat-induced genes and 17 heat-repressed genes in WT (in both biological repeats) (Fig. 2a, b; Additional file 1).

Similar to WT, only a small number of heat-repressed genes (17) were found in the *hrde*-*1* mutant animals. In contrast, 314 heat-induced genes were found in the *hrde*-*1* mutant animals (Fig. 2a, b; Additional file 1), a 8.5-fold increase over WT. These 314 genes include 26 of the 37 (70 %) heat-induced genes in WT (Fig. 2b; Additional file 1). The other 288 genes, referred to as ***n***uclear RNAi-repressed ***h***eat-inducible ***g***enes (NHGs) (Fig. 2b; Additional file 1), are likely to represent genes that are targeted by HRDE-1 in WT to prevent heat-induced gene activation.

We then compared mRNA levels of these NHGs in WT and *hrde*-*1* mutant at 23 °C, and found 41 of them indeed showed at least twofold HRDE-1-dependent repression in both repeats. In the *hrde*-*1* mutant, the heat-induced gene activation for these 41 NHGs is much stronger than the rest of NHGs, with a median ∆mRNA_[23 °C/15 °C]_ value of 8.0 (1.0 for WT). We refer to these 41 genes as high-stringent NHGs (Table 1).

**Table 1:** High-stringent nuclear RNAi-repressed heat-inducible genes (NHGs)

Gene name	Chr	Transposon	Note
*AC8.10*	X	MuDR transposable element	*AC8.10/11* and *AC8.3/4* are two duplicated neighboring sequences
*AC8.11*	X	MuDR transposable element	
*AC8.3*	X	MuDR transposable element	
*AC8.4*	X	MuDR transposable element	
*B0491.2.1* (*sqt-1*)	II	No obvious transposable elements around	*sqt-1* encodes a cuticle collagen; during larval and adult development, sqt-1 activity is required, likely redundantly, for normal cuticle, and hence organismal, morphology
*C13B9.4a.1* (*pdfr-1*)	III	A short CER9 LTR in an intron	*pdfr-1* encodes, by alternative splicing, three isoforms of a G-protein-coupled receptor (GPCR) required for normal locomotion
*C13B9.4b.1*	III	A short CER9 LTR in an intron	
*C13B9.4c.1*	III	A short CER9 LTR in an intron	
*C18D4.6a*	V	Several repeats, a short Tc1A at 3’ of the gene	
*C40A11.10*	II	Several repeats around the gene	
*C44B12.6*	IV	Several repeats around the gene	*C44B12.6* is an ortholog of human IL25 (interleukin 25), IL17C (interleukin 17C), IL17B (interleukin 17B), IL17D (interleukin 17D), and IL17A (interleukin 17A); *C44B12.6* is predicted to have cytokine activity, based on protein domain information
*C49C3.12* (*clec-197*)	IV	No obvious transposable elements around	C-type LECtin
*C50F4.3* (*tag-329*)	V	No obvious transposable elements around	*tag-329* is an ortholog of human CTSL (cathepsin L), CTSV (cathepsin V) and CTSO (cathepsin O); *tag-329* is predicted to have cysteine-type peptidase activity, based on protein domain information
*C52E2.6* (*fbxb-97*)	II	No obvious transposable elements around	F-box B protein
*F40D4.13*	V	CER15	
*F41G4.5*	X	CER12	
*F41G4.7*	X	CER12	
*F55D10.* *3* (*glit-1*)	X	No obvious transposable elements in the gene except some repeats around the gene	*glit-1* is an ortholog of human TG (thyroglobulin)
*F57G4.9*	V	Repeat Turmoil2, Haringer Family	This gene was determined to be of Transposon in origin so has been supressed from the *C. elegans* protein set
*F58H7.5*	IV	CER3 (gypsy)	
*F58H7.7*	IV	CER3 (gypsy)	
*F59B2.12*	III	No obvious transposable elements around	
*M7.9*	IV	No obvious transposable elements around	
*R09H3.3*	X	CER12-I ~ 4 kb downstream	
*T10D4.4* (*ins-31*)	II	Several repeats around the gene	*ins-31* encodes an insulin-like peptide of the insulin superfamily of proteins (OMIM: 176730, 147440); INS-31 is one of 38 insulin-like peptides in *C. elegans*, but is unique in that it contains three insulin repeats; although overexpression with INS-19 can result in low levels of larval lethality, the precise role of INS-31 in *C. elegans* development is not yet clear as INS-31/19 overexpression does not enhance dauer arrest in either a wild-type or *daf-2* mutant background, and loss of INS-31 function does not result in a mutant phenotype
*T11F9.10*	V	CER15	Paralog of *F40D.13*, both overlaps with CER15
*T11F9.18* (*srh-1*)	V	upstream to CER15	
*W08F4.9* (*fbxb-14*)	II	No obvious transposable elements in the gene except some repeats around the gene	*fbxb-14* (F-box B protein)
*Y37H2A.4* (*fbxa-107*)	V	CER3 ~ 500 bp upstream	F-box A protein. This gene encodes a protein containing an F-box, a motif predicted to mediate protein–protein interactions either with homologs of yeast Skp-1p or with other proteins; this gene’s encoded protein also contains an FTH/DUF38 motif, which may also mediate protein–protein interaction
*Y38H6* *C* *.5* (*dct-10*)	V	CER3-I in gene body, Tc4 right downstream	*dct-10* (DAF-16/FOXO Controlled, germline Tumor affecting), *dct-10* is predicted to have nucleic acid binding activity and zinc ion binding activity, based on protein domain information
*Y43F4A.3*	III	CER9	
*Y53F4B.10*	II	Several repeats around the gene	*Y53F4B.10* is orthologous to the human gene INSULIN (IRF4; OMIM: 176730), which when mutated leads to disease
*Y56A3A.5*	III	Several repeats in and around the gene	*faah-5* is an ortholog of human FAAH (fatty acid amide hydrolase) and FAAH2 (fatty acid amide hydrolase 2); faah-5 is predicted to have carbon–nitrogen ligase activity, with glutamine as amido-*N*-donor, based on protein domain information
*Y68A4A.13*	V	Several repeats in and around the gene	
*Y77E11A.3*	IV	Several repeats around the gene	
*Y79H2A.4*	III	Several Repeat around the gene	*Y79H2A.4* is an ortholog of human NDUFAB1 (NADH dehydrogenase (ubiquinone) 1, alpha/beta subcomplex, 1); *Y79H2A.4* is predicted to have nucleotide binding activity, based on protein domain information
*ZC15.1*	V	CER12-I downstream	
*ZC15.10*	V	CER12-I upstream	
*ZC15.3*	V	CER12	
*ZK262.8*	V	CER8	
*ZK262.9*	V	CER8	

By using the published HRDE-1-coIP small RNA dataset [10], we found that NHGs tend to have abundant HRDE-1-bound siRNAs (Fig. 2c), strongly suggesting that they are targeted by the germline nuclear RNAi pathway. We then examined the mRNA levels of 4 high-stringent NHGs (*F40D4.13*, *F41G4.7*, *ZK262.9*, and *F58H7.5*) in dissected gonads from adult WT or *hrde*-*1* mutant (23 °C) using qRT-PCR. The results confirmed that the desilencing in the *hrde*-*1* mutant occurs in the germline tissue for these genes (Additional file 2).

We then compared the mRNA-seq results between WT and *hrde*-*1* mutant (repeat 1) and found 72, 208, and 67 HRDE-1-repressed genes (∆mRNA_[*hrde*-*1/*WT]_ ≥ 2.0 [FDR ≤0.1]), at 15, 23, and p15 °C, respectively (Additional files 3, 4). Among the 208 HRDE-1-repressed genes at 23 °C, 72 genes overlap with the ones at 15 °C or p15 °C, representing a category of genes in which HRDE-1-dependent repression occurs at both 15 and 23 °C. This result also indicates that germline nuclear RNAi is active at both low and high temperature. The other 136 genes, which are unique to the 23 °C condition, represent a category in which HRDE-1-dependent repression occurs at 23 °C but not at 15 °C. Similar results were obtained with the second biological repeat (Additional files 3, 4).

We found that 34.1, 36.6, or 43.9 % of the 41 high-stringent NHGs overlap with LTR retrotransposon elements (approximately 0.4 % of the genome) with 0, 500, or 1000 bp flanking regions, respectively, representing a significant association (*p* values = 0, based on 100,000 Monte Carlo simulations, see “Methods”). This result evidences that a subset of LTR retrotransposons are heat inducible and that this temperature effect is normally prevented by HRDE-1. In contrast to LTR retrotransposons, only 2 % of the high-stringent NHGs overlap with DNA transposons (annotated as the Tc-type, approximately 0.5 % of the genome) with 1000 bp flanking sequences. A statistically significant association between the 288 NHGs and LTR retrotransposons was also found (see “Methods”).

In spite of the enrichment of LTR retrotransposons, the majority of the NHGs are protein-coding genes and do not appear to be associated with any transposon elements. We also examined the genomic distribution of NHGs in the arms (enriched for extensive heterochromatin domains) and the central region of each autosome (largely depleted for large heterochromatin domains) [19–21]. We found that NHGs are enriched in the arms of chromosomes I, II, and X and de-enriched in the central regions of chromosomes I and V (Fig. 2d, e). But these features in other chromosomes are not statistically significant (Fig. 2e). Therefore, nuclear RNAi maintains stable expression of a broad set of genes under heat stress. This ability is likely to be essential for *C. elegans* in its natural habitat where persisting heat stress is probably quite frequent [22, 23].

### Heat-induced gene activation of HRDE-1 targets occurs at the transcriptional level

**Fig. 3:**
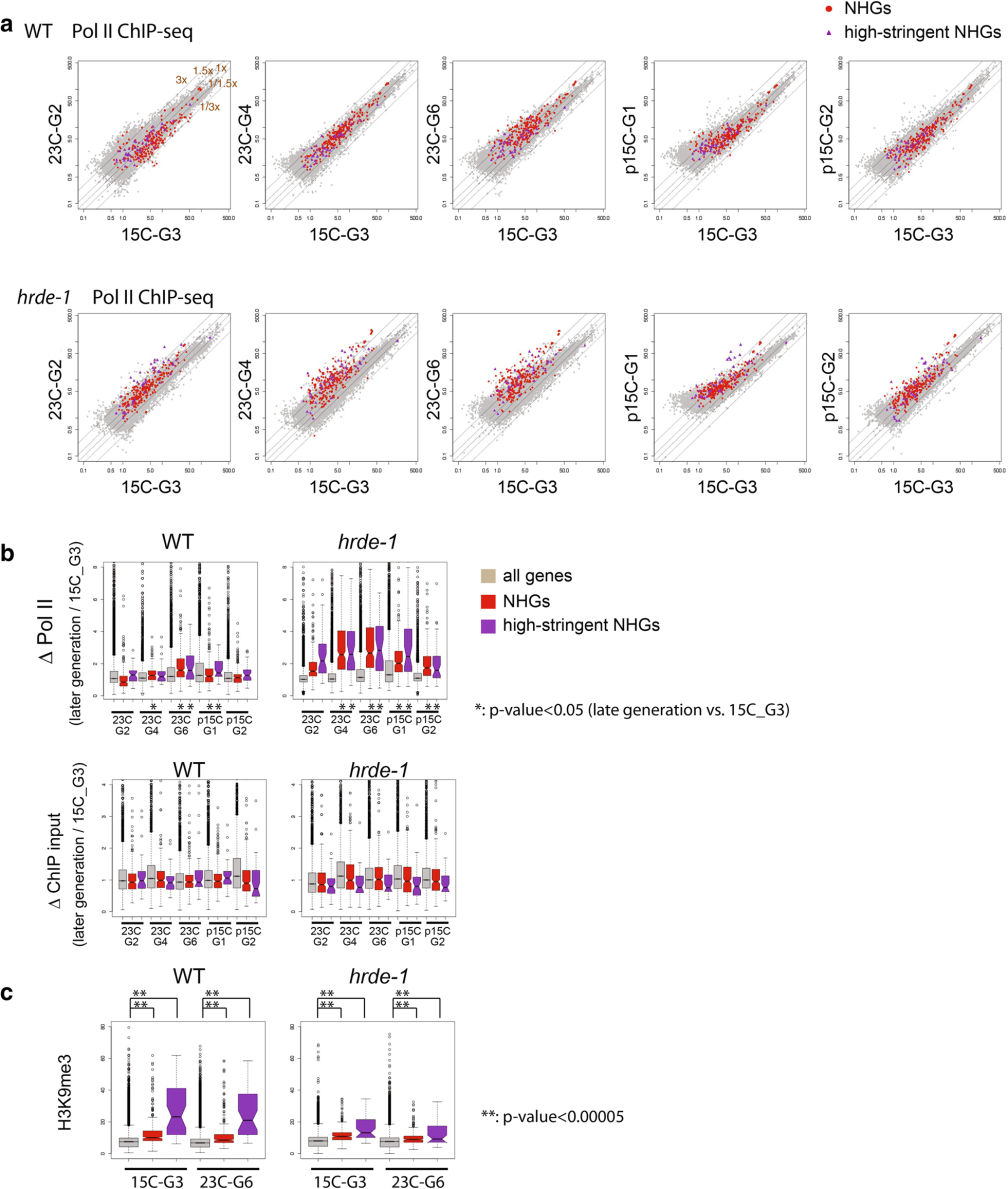
Heat-induced gene activation of HRDE-1 targets occurs at the transcriptional level. **a** Scatter plots comparing Pol II levels between 15C-G3 and each of 23C-G2, 23C-G4, 23C-G6, p15C-G1, and p15C-G2 for WT and *hrde*-*1* mutant. **b** Box plot analyses of changes (a later generation vs. 15C-G3) in Pol II levels for all protein-coding genes, NHGs, and high-stringent NHGs. Later generations with significant increases (*p* value <0.05, Wilcoxon rank-sum test) over 15C-G3 were indicated by “*.” **c** Box plot analysis indicating that NHGs, particularly the high-stringent ones, are associated with high levels of H3K9me3. Wilcoxon rank-sum test was used to calculate the *p* values

To test whether heat activation of NHGs occurs at the transcriptional level, we performed Pol II ChIP-seq and H3K9me3 ChIP-seq analyses for the 15C-G3, 23C-G2, 23C-G4, 23C-G6, p15C-G1, and p15C-G2 generations. The whole-genome Pol II profile at each of the three 23 °C-generations (23C-G2, 23C-G4, or 23C-G6) was compared with the one at the 15C-G3. For most of the genes in the *C. elegans* genome, the Pol II levels at the 15C-G3 were similar to the 23 °C-generations (Fig. 3a, b). This is the case for both WT and *hrde*-*1* mutant. However, in the *hrde*-*1* mutant, NHGs exhibited significantly higher Pol II levels at the 23 °C-generations than the ones at 15 °C (Fig. 3a, b). The median values of ∆Pol II_[23C-G2/15C-G3]_, ∆Pol II_[23C-G4/15C-G3]_, and ∆Pol II_[23C-G6/15C-G3]_ for the 288 NHGs were 1.52, 2.55, and 2.65, respectively, in the *hrde*-*1* mutant, significantly higher than the ones in the wild type (0.84, 1.26, and 1.59, respectively). NHGs in WT also showed increased Pol II levels in the 23C-G4 and 23C-G6 generations compared to the 15C-G3, but the effect was much weaker than the *hrde*-*1* mutant (Fig. 3a, b). These results indicate that (1) some of the germline nuclear RNAi target genes are susceptible to heat-induced transcriptional activation, and (2) this temperature effect is normally prevented by the germline nuclear RNAi pathway.

Similar analysis for the H3K9me3 ChIP-seq assay revealed that the 23 °C-generations samples had a modest but consistent reduction of H3K9me3 levels for NHGs in both the wild-type and the *hrde*-*1* mutant animals when compared with the 15C-G3 sample (Additional file 5). We also found that NHGs genes are associated with high levels of H3K9me3 in both the wild-type and *hrde*-*1* mutant animals (Fig. 3c).

### Transgenerational effect of temperature on gene expression in the *hrde*-*1* mutant

While analyzing the Pol II ChIP-seq data, we noticed that the heat-induced transcriptional activation of NHGs is stronger in 23C-G4, and 23C-G6 than 23C-G2 (Fig. 3b). The median values of ∆Pol II_[23C-G4/23C-G2]_ and ∆Pol II_[23C-G6/23C-G2]_ for NHGs in the *hrde*-*1* mutant are 1.6 and 1.7, respectively, (*p* values <1 × 10^−8^ for both, Wilcoxon rank-sum test). In addition, heat-induced transcriptional activation persisted when *hrde*-*1* mutant was shifted back to 15 °C. The median values of ∆Pol II_[p15C-G1/15C-G3]_ and ∆Pol II_[p15C-G2/15C-G3]_ for NHGs in the *hrde*-*1* mutant are 2.0 and 1.7, respectively, (*p* values <1 × 10^−12^ for both). We consider both types of effects to be transgenerational, namely, the intensified derepression in the late heat stress generations over the early one and the persistent derepression in the p15C generations. These transgenerational effects are detectable in WT as well, but much less pronounced than in the *hrde*-*1* mutant. For example, the median values of ∆Pol II_[p15C-G1/15C-G3]_ and ∆Pol II_[p15C-G2/15C-G3]_ for NHGs in WT are 1.2 (*p* value = 0.002) and 1.1 (*p* value = 0.2), respectively. We found that the effect of heat stress on H3K9me3 for the NHGs is transgenerational as well (Additional file 5). Similarly to Pol II, the transgenerational effect of on H3K9me3 is more pronounced in the *hrde*-*1* mutant than the WT animals. These results indicate that (1) temperature-induced chromatin effect can be transgenerationally heritable and (2) this transgenerational effect is normally repressed by the germline nuclear RNAi pathway.

**Fig. 4:**
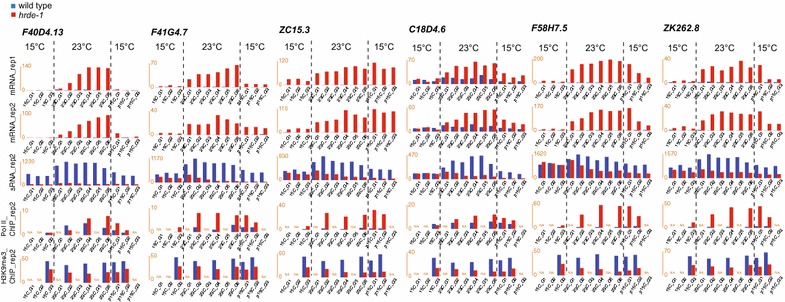
Exemplary cumulative NHGs with their temporal profiles of mRNA (12 generations, two biological repeats), endo-siRNA (12 generations), Pol II (6 generations), and H3K9me3 (6 generations)

To identify genes with heat stress-induced transgenerational effect, we examined the 12-generation mRNA expression profiles of the high-stringent NHGs (Additional file 6). Among these genes, six showed a trend of progressive increase in their mRNA expression levels from 23C-G1 to 23C-G6 (Fig. 4) in the *hrde*-*1* mutant. We will refer to these genes as exemplary cumulative NHGs. Most of these cumulative NHGs are within or in close proximity of retrotransposon elements (e.g., *F40D4.13, F41G4.7, ZC15.10*, and *F58H7.5*). mRNA expression of these cumulative NHGs in WT is largely repressed at both 15 and 23 °C (Fig. 4).

For some of the cumulative NHGs (e.g., *ZC15.3, F58H7.5,* and *C18D4.6*), the heat-induced gene activation persisted for 2–3 generations after the *hrde*-*1* mutant animals were shifted back to 15 °C (Fig. 4). The Pol II levels of the cumulative NHGs showed a transgenerational effects that are generally consistent with the corresponding mRNA changes, indicating that the transgenerational effect occurs at the transcriptional level (Fig. 4). These cumulative NHGs evidenced that gene expression programs can be influenced not only by the current environment, but also the environment experienced by ancestors. Because these cumulative/transgenerational effects occurred in the *hrde*-*1* mutant but not WT, we suggest that one of the HRDE-1-dependent RNAi functions is to prevent the transgenerational effect of the heat-induced transcriptional activation, which may result in feed-forward amplification of unwanted gene activation and reduced fitness at the organismal level.

### Transgenerational effect of heat stress on siRNA expression

To investigate the effects of *hrde*-*1* mutation and temperature on siRNA expression at the multigenerational time scale, we performed small RNA-seq experiments for one set of the temperature-shift samples (12 generations for WT and *hrde*-*1*[*tm1200*]). The 5′-mono-phosphate (mono-Pi)-independent small RNA cloning procedure was used to capture both the primary and secondary siRNAs.

**Fig. 5:**
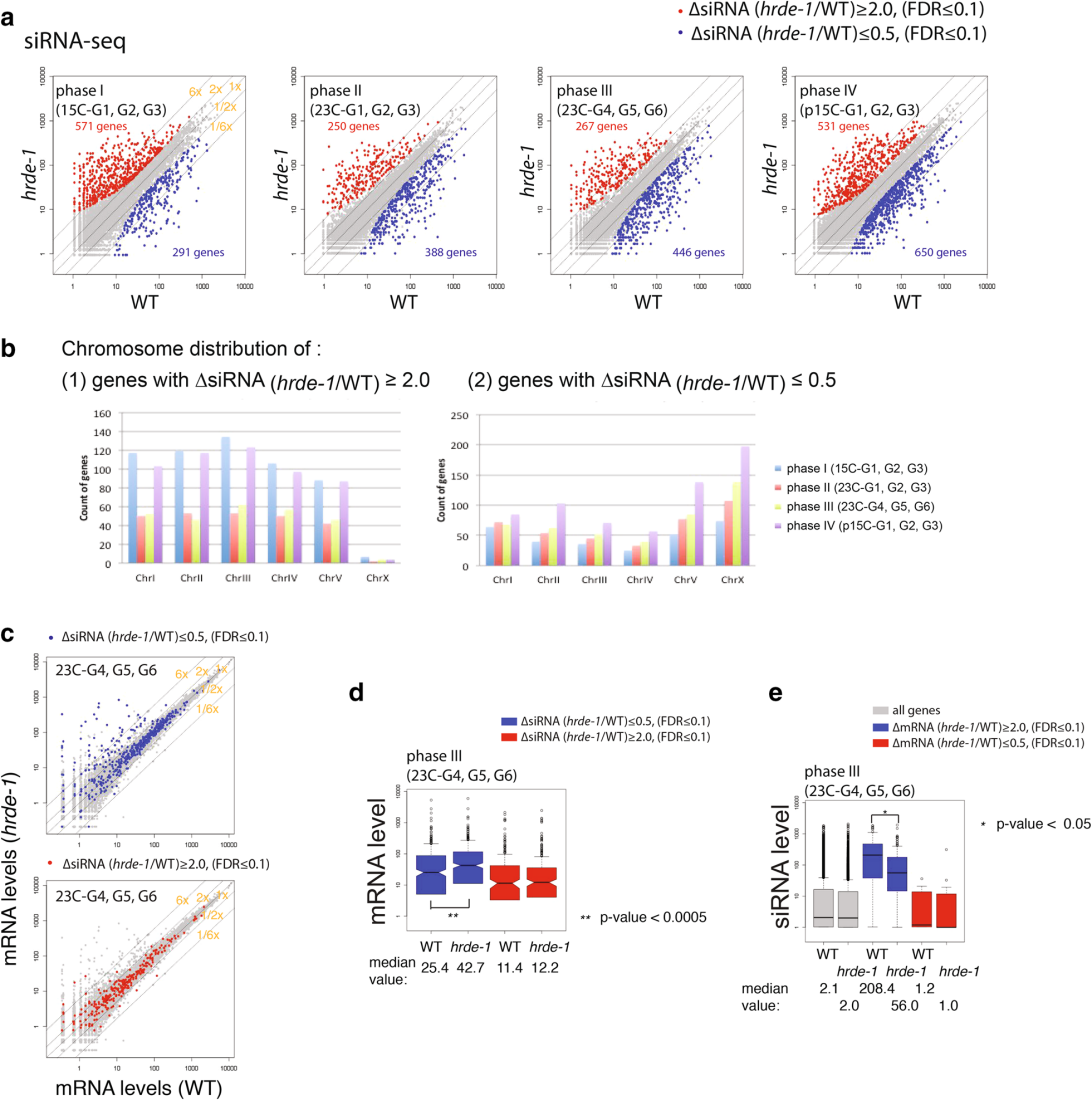
Endo-siRNA changes caused by *hrde*-*1* mutation at different temperatures and generations. **a** Scatter plots comparing endo-siRNA levels of WT and *hrde*-*1* mutant. The 12 generations were divided evenly into 4 phases (I to IV) chronologically. The normalized mean siRNA counts of three generations in each phase were used in this analysis for each protein-coding gene. Genes with increased siRNAs in the *hrde*-*1* mutant over WT at each phase were highlighted in *red* (*blue* for ones with decreased siRNAs). **b** Chromosome distribution of genes with HRDE-1-dependent siRNA expression changes. **c** Scatter plots comparing mRNA levels of WT and *hrde*-*1* mutant with genes with *hrde*-*1*(-)-dependent siRNA changes highlighted. **d** Box plot comparing mRNA expression levels between WT and *hrde*-*1* mutant for genes with *hrde*-*1*(-)-dependent siRNA changes. **e** Box plot comparing siRNA expression between WT and *hrde*-*1* mutant for all genes and genes with *hrde*-*1*-dependent mRNA changes. Only phase III (23C-G4, G5, and G6) results were plotted in *panels* **c**–**e**. See Additional file 7 for results of all four phases. Wilcoxon rank-sum test was used to calculate the *p* values

To examine the effect of *hrde*-*1* mutation on siRNA expression, we divided the 12 generations into four phases chronologically (phase I: 15C-G1, 15C-G2, and 15C-G3; phase II: 23C-G1, 23C-G2, and 23C-G3; phase III: 23C-G4, 23C-G5, 23C-G6; and phase IV: p15C-G1, p15C-G2, p15C-G3). The average number of small RNAs that are anti-sense to mRNA for each gene was calculated for each phase. By using a minimal fold-of-change of 2.0 (FDR ≤0.1) as cutoff, we found 250–571 genes with increased siRNA expressions and 291–650 genes with decreased siRNA expression in the *hrde*-*1* mutant over WT in the 4 phases (Fig. 5a).

We examined the relationship between *hrde*-*1(*-*)*-dependent siRNA changes and *hrde*-*1(*-*)*-dependent mRNA changes. We found a significant fraction (31–47 %) of HRDE-1-repressed genes at 15 or 23 °C have reduced siRNA expressions in the *hrde*-*1* mutant over WT at the corresponding temperature (Fig. 5c, e; Additional file 7). However, we note that the majority of the genes with siRNA changes (either increase or decrease in *hrde*-*1* mutant) have similar mRNA expressions between the *hrde*-*1* mutant and WT in each of the four phases (Fig. 5c, d, e; Additional file 7), suggesting that the connection between the siRNA and mRNA expression changes (WT v.s. *hrde*-*1* mutant) is complex and may be limited. siRNAs of germline-specific genes (based on [24]) and CSR-1 target genes (based on the CSR-1-coIP-siRNA profile from [25]) are not affected by *hrde*-*1* mutation (Additional file 8). In contrast, a large number of HRDE-1 target genes (based on the HRDE-1-coIP-siRNA profile from [10]) exhibited siRNA expression changes (either increase or decrease) in the *hrde*-*1* mutant (Additional file 8).

Interestingly, we found that genes with increased siRNA expression in the *hrde*-*1* mutant are severely depleted in the X chromosome (5.6–27.6 fold lower than the expected frequency if random distribution is assumed, *p* value = 0, Monte Carlo simulation) and approximately evenly distributed in five autosomes (Fig. 5b; Additional file 7d).

We also noticed that the numbers of genes with decreased siRNA expression in the *hrde*-*1* mutant progressively increases from phase I to phase IV (Fig. 5b and Additional file 9). We performed a Venn diagram analysis and found that this trend is due to (1) new genes joined this category in each of phases II, III, and IV, and (2) genes falling into this category in the previous phase tend to remain in the category in all later phases (data not shown). The phase IV has the highest number of such genes despite the fact that the temperature was shifted back to 15 °C, suggesting a transgenerational cumulative effect of heat stress on siRNA expression.

We then examined the 12-generation siRNA profiles for the high-stringent NHGs individually, and found that for some genes, the *hrde*-*1* mutant and WT showed opposite effects of temperature on siRNA expression (e.g., *F41G4.7, ZC15.3, C18D4.6,* and *F58H7.5*) (Fig. 4). While heat stress leads to increases in siRNA levels of these cumulative NHGs in WT, it leads to decrease in the *hrde*-*1* mutant, suggesting a role of siRNAs in responding to the temperature change and repressing heat-inducible elements. Interestingly, the temperature effects on some NHGs in the *hrde*-*1* mutant appear to be transgenerational progressive at 23 °C (e.g., *F41G4.7, C18D4.6*, and *F58H7.5*) (Fig. 4). In addition, while the changes in siRNAs at 23 °C-generations in the wild-type animals were reverted in the post-23 °C 15 °C-generations, the temperature effect on siRNAs at 23 °C-generations in the *hrde*-*1* mutant animals persisted in the post-23 °C 15 °C-generations (e.g., *F41G4.7, ZC15.C, C18D4.6,* and *F58H7.5*) (Fig. 4). For some genes, the siRNA profiles mirror nicely with their mRNA profiles over the 12 generations, suggesting that the transgenerational effects of temperature on siRNA and mRNA expression may be tightly linked in these cases.

## Discussion

The germline of animals, including human, exhibits much higher temperature sensitivity than somatic tissues. Small temperature increases by a few degrees can sometimes disrupt the gamete functions or the process of gametogenesis [26–29]. This is a greater challenge for invertebrates such as *C. elegans,* which cannot control its body temperature, yet experiences a large degree of temperature fluctuation in their natural environment [22, 23]. In this 12-generational temperature-shift study, we found that the impact of an 8 °C-temperature change on the transcriptome of the WT *C. elegans* strain is very limited. (We note that the two temperature points used in this study, 15 and 23 °C, are within the range of permissive temperature for WT *C. elegans.*) For a large number of genes, the thermal stability of mRNA expression requires the germline nuclear AGO protein HRDE-1. These results revealed that genomic regions with intrinsic temperature sensitivity are more prevalent than previously recognized. Furthermore, transcription activities in these regions are actively monitored and repressed in *C. elegans* germline. Several features of the nuclear RNAi pathway make it well suited to perform these tasks. (1) siRNAs can function both as sensor to reflect transcriptional changes and as guide molecules to silence the target genes. (2) The chromatin-based silencing mechanism leads to stable gene repression. (3) Both siRNA and H3K9me3 can function as epigenetic memory for transgenerational gene silencing. RNAi and various RNA-mediated chromatin regulation mechanisms in mammals share these features, and may play similar roles.

Our previous study showed that LTR retrotransposons constitute a major class of native targets of germline nuclear RNAi in *C. elegans* [16]. Here, we showed that a significant fraction of these retrotransposons are heat inducible. Transposon activation has been linked to environmental factors, various forms of stress in particular, in bacteria, plants, and animals [30–32]. The transposon-host genome interactions have been proposed as a co-evolution mechanism to generate novel genetic variability that allows host or transposon, or both, to better cope with stress. We note that not all LTR retrotransposons in *C. elegans* are targeted by nuclear RNAi for silencing. *cer1*, a *gypsy*-type LTR retrotransposon, is transcriptionally active and produces virus-like particle in the germline of WT *C. elegans* [33]. In this study, we showed that transcriptional regulation of many germline nuclear RNAi native targets, including some of the LTR retrotransposons, is independent of temperature. Germline nuclear RNAi is required at both low and high temperature to silence these targets. The difference in temperature responses may be due to the difference in sequence/structure of the transposons or nearby host genes. Further study is needed to determine whether the LTR retrotransposons can mutate the host genome via transposition when they are transcriptionally activated in the germline nuclear RNAi mutants.

We also note that the profile of HRDE-1-coIP siRNAs suggests that many endogenous protein-coding genes can be potentially targeted by germline nuclear RNAi pathway. Most of these genes do not show transcriptional derepression or reduced H3K9me3 when worms are under heat stress (or at the permissive temperature) in the germline nuclear RNAi mutants [16]. It is possible that the germline nuclear RNAi is required to repress these genes under another kind of environmental stress.

Recent studies have demonstrated that transgenerational epigenetic mechanisms are not limited to gene silencing. For example, active germline expression of *fer*-*1,* a *C. elegans* germline-specific gene, and certain transgenes is dependent on the active germline expression state in the previous generation, a phenomenon termed as germline licensing effect or RNAa [34, 35]. Although the underlying mechanisms are largely unknown, siRNAs, particularly CSR-1-associated ones, have been linked to these phenomena [35–37]. In this study, we showed that the heat-induced aberrant transcription in the *hrde*-*1* mutant can be heritable and some of these effects can even cumulate from one generation to the next. These findings suggest a potential vulnerability of the germline licensing effect, which may contribute the *Mrt* phenotype of the germline nuclear RNAi mutants. The germline nuclear RNAi pathway may have evolved to balance the germline licensing effect and maintain transgenerational homeostatic gene expression in a fluctuating environment. Alternatively, heat stress-induced transcription activation observed in the *hrde*-*1* mutant is independent of the germline licensing pathway, but instead due to loss of chromatin-based silencing. Further studies are needed to investigate these possibilities.

## Conclusions

We investigated the impact of heat stress on mRNA expression, transcription, H3K9me3, and siRNAs at the whole-genome level in the wild-type *C. elegans* and a mutant strain that lacks the germline-specific nuclear AGO protein HRDE-1/WAGO-9. In contrast to a small number of heat-induced genes in the WT, many genes (>280) become activated by heat in the *hrde*-*1* mutant. These genes have abundant HRDE-1-associated siRNAs and high levels of HRDE-1-dependent H3K9me3. Our study expands the list of genes that are regulated by the germline nuclear RNAi pathway and indicates a previously unrecognized contribution of this pathway to the stability of germline gene expression in a changing environment. Furthermore, we demonstrated that the heat-induced gene activation of germline nuclear RNAi target genes occur at the transcriptional level. In some loci, the heat-induced transcriptional activation is transgenerationally heritable in the *hrde*-*1* mutant. A subset of LTR retrotransposons are particularly sensitive to this transgenerational epigenetic effect. siRNA expression in these regions tend to be heat inducible in the wild-type animals, but not in the *hrde*-*1* mutant. Together, our findings establish that the effect of temperature on epigenome and gene expression in *C. elegans* germline is closely monitored by small RNA pathways, and point to a physiologically relevant role of nuclear RNAi in maintaining an immortal germline by repressing deleterious epigenetic effects induced by environmental stress.

## Methods

### Multigenerational temperature-shift experiment

*C. elegans* strain N2 and *hrde*-*1 (tm1200)* were cultured on NGM plates with *E. coli* OP50 as the food source in a temperature controlled incubator [38]. Worms were maintained at 15 °C prior to the multigenerational temperature-shift experiment.

For the multigenerational temperature-shift experiment, synchronized worms were cultured at 15 °C for three generations (15C-G1, G2, and G3), and then at 23 °C for six generations (23C-G1 to G6), followed by 15 °C for three generations (p15C-G1, G2, and G3). For synchronization, eggs were harvested by using the hypochlorite bleaching method described in [39]. Synchronized L1 population was obtained by placing embryos on unseeded NGM plates for 16–24 h. L1s were then released onto 10-cm NGM plates with OP50 (approximately 4500 L1s per plate) at each generation. Ten L1s from each generation were single picked for the brood-size assay. For the 15 °C-generations (15C-G1-G3 and p15C-G1-G3), young adults were collected 114–120 h after L1 s were released. For the 23 °C-generations (23C-G1-G6), young adults were collected 50–55 h after L1 s were released. All reagents used here were pre-warmed to 15 or 23 °C depending on the target temperature at each generation. For the 23C-G1 and p15C-G1 samples, temperature shift began at the embryogenesis step. Young adults were pulverized by grinding in liquid nitrogen with a mortar and pestle immediately after sample harvest and were stored at −80 °C. Ground worms were used for all assays in this study.

### High-throughput sequencing (HTS)

mRNA-seq: For each sample, total RNA was extracted from approximately 5000 worms using Trizol reagent (Life Technologies). mRNA was enriched using the Poly(A) Purist MAG kit (Life Technologies). 0.5–1 μg of mRNA was used to prepare each mRNA-seq library as described in [16].

ChIP-seq: Approximately 5000 worms were used for each chromatin immunoprecipitation experiment according to the procedure described in [16]. Anti-H3K9me3 (ab8898, Abcam) and anti-RNA polymerase II CTD repeat YSPTSPS (phosphor-S2) (ab5095, Abcam) antibodies were used for the H3K9me3 and Pol II ChIP, respectively. These ChIP experiments usually yielded 5–10 ng DNA. The entire ChIP DNA or 10 ng DNA in the case of ChIP input was used to make DNA library with the KAPA Hyper Prep Kit (KAPA Biosystems) according to the manufacturer’s instruction.

sRNA-seq: Small RNA was enriched using the mirVana™ miRNA Isolation Kit (Life Technologies). Small RNA library was prepared from 1 µg small RNA by using a 5′-mono-phosphate-independent small RNA cloning procedure described in [40].

Multiplexing: A total of 108 DNA libraries were prepared for this study. Each library is barcoded with a unique 6-mer index located on the 3′ linker. Libraries of the same type (mRNA-seq, sRNA-seq, or ChIP-seq) and the same biological repeat were pooled together for HTS.

HTS instrument: Pooled libraries were sequenced on an Illumina HiSeq 2500 platform with the following specifications: rapid run mode, 50-nt single-end run, and index sequencing. De-multiplexed raw data in fastq format were provided by the sequencing service provider. The average numbers of sequenced tags per library are 4.6, 5.3, and 3.1 million for mRNA, small RNA, and ChIP-seq, respectively.

Data availability: De-multiplexed raw sequencing data in fastq format for all 108 libraries were deposited in NCBI (GEO accession number: GSE74405).

### Bioinformatics

Sequence alignments were performed by using the software Bowtie (version 0.12.7) [41]. The *C. elegans* genomic sequence (WS190 release) was used to align the ChIP-seq reads. Alignments to both sense and anti-sense strands of the reference genome were used. The mRNA database (WS190) was used to align the mRNA-seq and small RNA-seq reads. Only perfect alignments were used for the subsequent analysis. For mRNA-seq analysis, only the sense-strand alignments were used. For sRNA-seq analysis, only the anti-sense alignments were used.

Identifying temperature-sensitive (ts) genes: The Bioconductor R package of DEseq [42] was used for this analysis. The 15C-G1, 15C-G2, and 15C-G3 samples were used to calculate the mean mRNA expression levels at 15 °C for wild type or *hrde*-*1* mutant; the 23C-G2, 23C-G3, 23C-G4, 23C-G5, and 23-G6 samples were used to calculate the mean mRNA expression levels at 23 °C. The 23C-G1 samples were excluded from this analysis because the 15-to-23 °C transition occurred during the embryogenesis stage for these samples. The p15C-G1, p15C-G2, and p15C-G3 samples were excluded from this analysis to avoid any potential transgenerational effect of the previous exposure to 23 °C. Heat-induced genes were defined as genes with at least twofold increase in their mean mRNA expression levels at 23 °C over the ones at 15 °C (FDR ≤0.1) in both biological repeats. Heat-repressed genes were defined as genes with at least twofold decreases in their mean mRNA expression levels at 23 °C over the ones at 15 °C (FDR ≤0.1) in both biological repeats.

RPKM value for ChIP-seq analysis was defined as the number of reads mapped to a gene normalized by the total reads in the experiments (in millions) and the length of the gene (in kilo base-pairs) using Python scripts. RPKM values for mRNA and sRNA-seq analysis were similarly defined except using the exon length for a gene for the normalization.

### Statistics

One-sided Wilcoxon rank-sum test (R) was used to determine whether the two sets of data are significantly different. For Fig. 2c, RPKM values were used to compare the HRDE-1-bound siRNAs between non-NHGs and NHGs and between non-NHGs and high-stringent NHGs. For Fig. 3b, RPKM values of 15C-G3 and a later generation were used to compare the Pol II levels between the two datasets. For Fig. 3c, RPKM values were used to compare the H3K9me3 levels between all genes and NHGs and between all genes and high-stringent NHGs.

Test the association between NHGs and LTR retrotransposon elements. Genomic locations for a total of 405,858 bp sequences that are annotated as LTR retrotransposon-type repeats (approximately 0.4 % of the *C. elegans* genome) were obtained from the UCSC genome. 4.9, 5.9, and 8.3 % of the 288 NHGs overlap with these LTR retrotransposon elements or the flanking regions (0, 500, and 1000 bp, respectively). To evaluate the significance of the association, we randomly chose 288 2.92-kb (the average size of the NHGs) regions and calculated the fraction (*F*) of these 288 regions that overlap or near LTR retrotransposon elements. This simulation was performed 100,000 times. The frequencies of the simulations with *F* ≥ 0.049, 0.059, or 0.083 (for 0, 500, or 1000 bp flanking sequence, respectively) were calculated (all less than 0.0002) and used to estimate the *p* values. *p* values for the 41 high-stringent NHGs were similarly calculated (all zeros).

### qRT-PCR

Total RNA was isolated from hand-dissected young gravid adult gonad by Trizol reagent (Life Technologies). To remove DNA contamination, total RNA was treated with DNase I (New England Biolabs) according to the manufacturer’s instructions and was then purified by Phenol: Chloroform extraction. 30 ng total RNA (30 ng) was used for reverse transcription using SuperScript III Reverse transcriptase (Life Technologies) and Oligo dT (Integrated DNA technologies). qPCR was performed using KAPA SYBR FAST Universal 2× PCR Master Mix (KAPA Biosystems) on a Mastercycler EP Realplex real-time PCR system (Eppendorf) according to the manufacturer’s instructions. qPCR primer sequences used in this study are as follows:

- F40D4.13 Forward: TGAGAGCTCAAAGCAAACGA
- F40D4.13 Reverse: AGACTCTCGCCAAGCATTGT
- F41G4.7 Forward: TGGGCTCAATTCAAGGAAAG
- F41G4.7 Reverse: CGACGTCTCCCTCTCTATGG
- ZK262.9 Forward: CGTACGTGATTTCGGAGGAT
- ZK262.9 Reverse: CTGCGCAGAAGACTTCATTG
- F58H7.5 Forward: AATGGCTCAAAATCCAGTCG
- F58H7.5 Reverse: GGTGTCACGAATCGTTGATG
- ges-1 Forward: GAAAACCGGCAGAAGTGAAG
- ges-1 Reverse: AGAGCCTCTTGCTTGCTCTG
- glp-1 Forward: CTCAAAATGAATGCGCAGAA
- glp-1 Reverse: TATCCCGAGTCGCATACACA
- pgl-1 Forward: TGTTGAGCTCACGGAACTTG
- pgl-1 Reverse: GATCGGCAGGTTCAGATTTC
- cdc-42 Forward: CTGCTGGACAGGAAGATTACG
- cdc-42 Reverse: CTCGGACATTCTCGAATGAAG

## Additional files

10.1186/s13072-016-0052-x Lists of genes with at least twofold changes (FDR ≤ 0.1) in mRNA expression between 23˚C (23C-G2 to G6) and 15˚C (15C-G1 to G3). Genes in various classes of the Venn diagram shown in Fig. 2b were also listed.
10.1186/s13072-016-0052-x qRT-PCR analyses using dissected gonads from adult WT or *hrde*-*1* mutant (23 ˚ C). mRNA levels of two germline-specific genes (*glp*-*1* and *pgl*-*1*), an intestine-specific gene (*ges*-*1*), and four high-stringent NHGs (*F40D4.13, F41G4.7, ZK262.9*, and *F58H7.5*) were examined for each of the samples.
10.1186/s13072-016-0052-x HRDE-1-repressed genes at different temperatures. (a) Scatter plots comparing the WT and *hrde*-*1* mutant transcriptomes at 15˚C, 23˚C, and p15˚C. (b) Venn diagram analysis of HRDE-repressed genes at different temperatures.
10.1186/s13072-016-0052-x Lists of HRDE-1-repressed genes (∆mRNA _[*hrde*-*1/*WT]_ ≥ 2.0 [FDR ≤ 0.1]) at 15˚C, 23 °C, and p15 °C.
10.1186/s13072-016-0052-x Box plot analyses of changes (later generations vs. 15C-G3) in Pol II for all protein-coding genes, NHGs, and high-stringent NHGs.
10.1186/s13072-016-0052-x Multigenerational mRNA, siRNA, Pol II, and H3K9me3 levels for the 41 high-stringent NHGs.
10.1186/s13072-016-0052-x Relationship between *hrde*-*1*-dependent siRNAs changes and *hrde*-*1*-dependent mRNA changes. (a) Scatter plots comparing mRNA levels of WT and *hrde*-*1* mutant for genes with *hrde*-*1*-dependent siRNA changes highlighted for all four phases. (b) Box plot comparing mRNA expression levels between WT and *hrde*-*1* mutant for genes with *hrde*-*1*-dependent siRNA changes for all phases. (c) Box plot comparing siRNA expression between WT and *hrde*-*1* mutant for all genes and genes with *hrde*-*1*-dependent mRNA changes. (The results for phase III [23C-G4, G5, and G6] in panels a, b, and c were also shown in Fig. 5c, 5d, and 5e.) (d) Genomic distributions of genes with *hrde*-*1*-dependent siRNA changes in all four phases. Wilcoxon rank-sum test was used to calculate the p-values.
10.1186/s13072-016-0052-x (a) Scatter plots comparing siRNA levels of WT and *hrde*-*1* mutant with germline-specific genes, CSR-1 targets, or HRDE-1 targets highlighted. (b) Box plots comparing siRNA levels of WT and *hrde*-*1* mutant for germline-specific genes, CSR-1 targets, or HRDE-1 targets. Germline specific genes were defined by [24]. CSR-1 or HRDE-1 target genes were defined by using the published CSR-1-coIP [25] or HRDE-1-coIP siRNA profiles [10], respectively. CSR-1 targets: ∆siRNA (CSR-1-coIP/HRDE-1-coIP) > 10 and CSR-1-coIP siRNA (rpkm) > 40. HRDE-1 targets: ∆siRNA (HRDE-1-coIP/CSR-1-coIP) > 10 and HRDE-1-coIP siRNA (rpkm) > 40.
10.1186/s13072-016-0052-x Stacked bar graph showing the accumulation of genes with siRNA reductions in the *hrde*-*1* mutant.

## Abbreviation

NHG: nuclear RNAi-repressed heat-inducible gene
