# Supplementary material for: A transgenerational role of the germline nuclear RNAi pathway in repressing heat stress-induced transcriptional activation in C. elegans
Source: Epigenetics Chromatin. 2016 Jan 15;9:3. doi: 10.1186/s13072-016-0052-x (PMC4714518; doi:10.1186/s13072-016-0052-x)

qRT-PCR high-stringent NHGs in dissected gonad of 23C-F6 generation

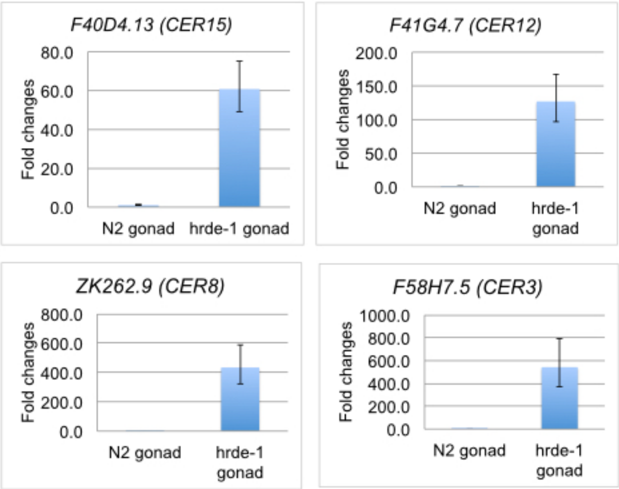

qRT-PCR intestine- and germline- specific genes in dissected gonads or intestines of 23C-F6 generation

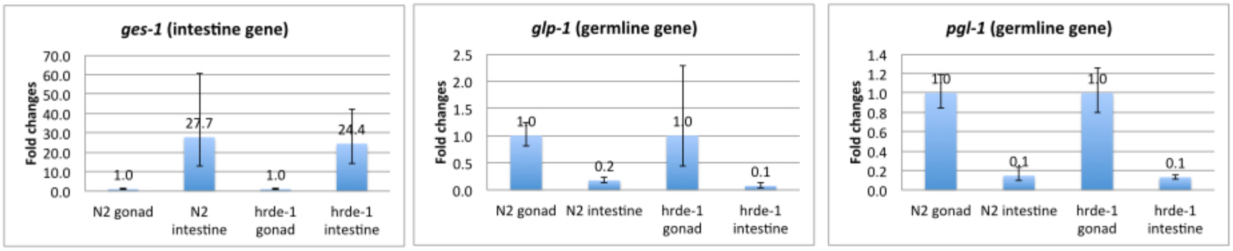

Supplement: Supplementary file 2 — 10.1186/s13072-016-0052-x qRT-PCR analyses using dissected gonads from adult WT or hrde-1 mutant (23 ˚ C). mRNA levels of two germline-specific genes (glp-1 and pgl-1), an intestine-specific gene (ges-1), and four high-stringent NHGs (F40D4.13, F41G4.7, ZK262.9, and F58H7.5) were examined for each of the samples. [file 13072_2016_52_MOESM2_ESM.pdf]
