# Supplementary figures and images for: A transgenerational role of the germline nuclear RNAi pathway in repressing heat stress-induced transcriptional activation in C. elegans
Source: Epigenetics Chromatin. 2016 Jan 15;9:3. doi: 10.1186/s13072-016-0052-x (PMC4714518; doi:10.1186/s13072-016-0052-x)

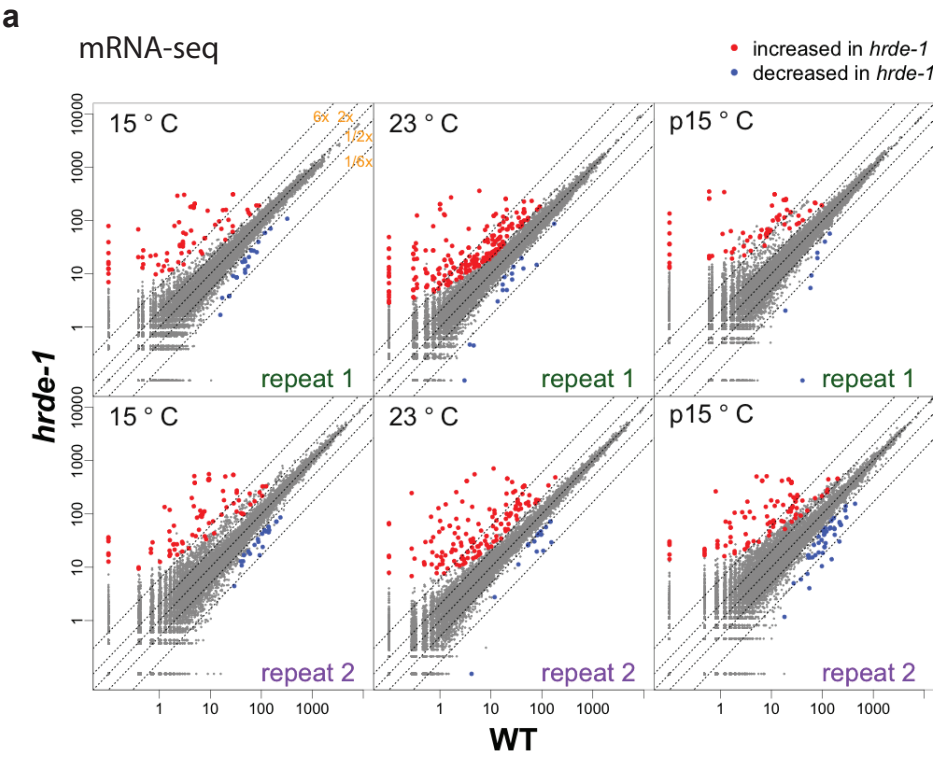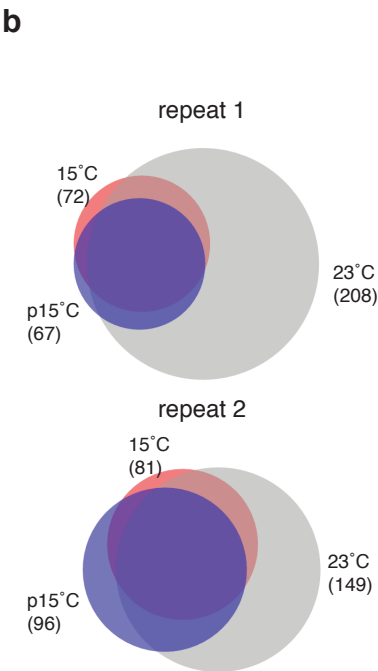

Supplement: Supplementary file 3 — 10.1186/s13072-016-0052-x HRDE-1-repressed genes at different temperatures. (a) Scatter plots comparing the WT and hrde-1 mutant transcriptomes at 15˚C, 23˚C, and p15˚C. (b) Venn diagram analysis of HRDE-repressed genes at different temperatures. [file 13072_2016_52_MOESM3_ESM.pdf]

$\Delta$  H3K9me3  
(later generation / 15C\_G3)

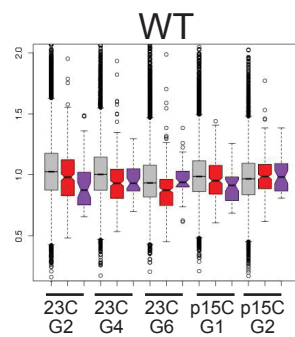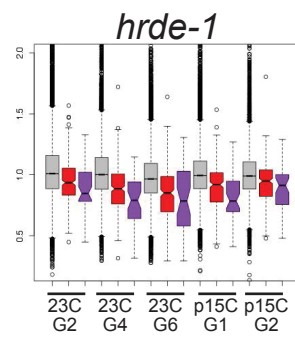

- all genes
- NHGs
- high-stringent NHGs

Supplement: Supplementary file 5 — 10.1186/s13072-016-0052-x Box plot analyses of changes (later generations vs. 15C-G3) in Pol II for all protein-coding genes, NHGs, and high-stringent NHGs. [file 13072_2016_52_MOESM5_ESM.pdf]

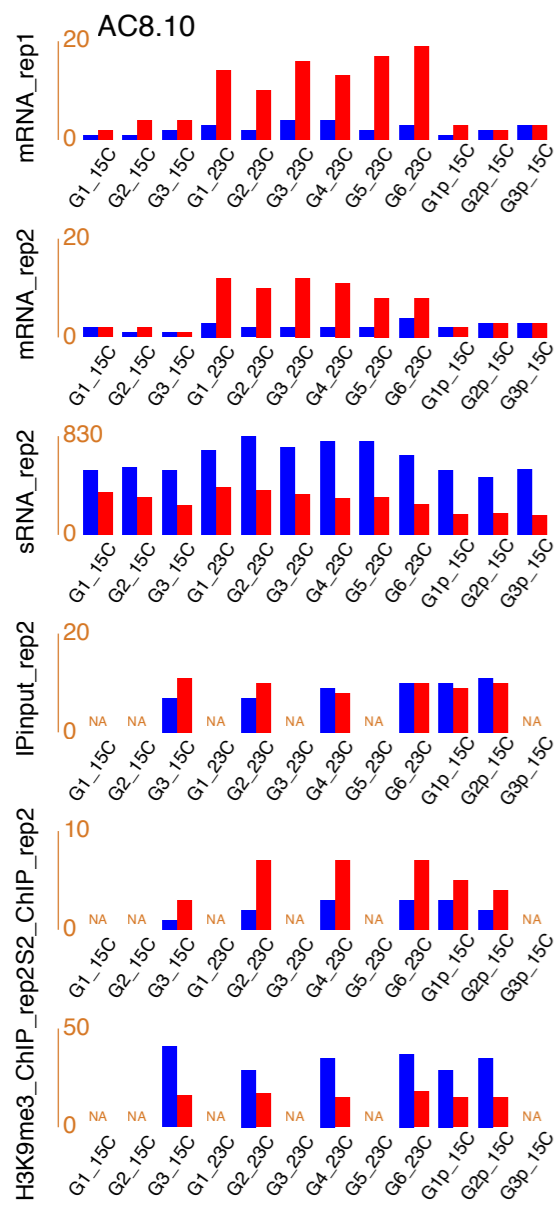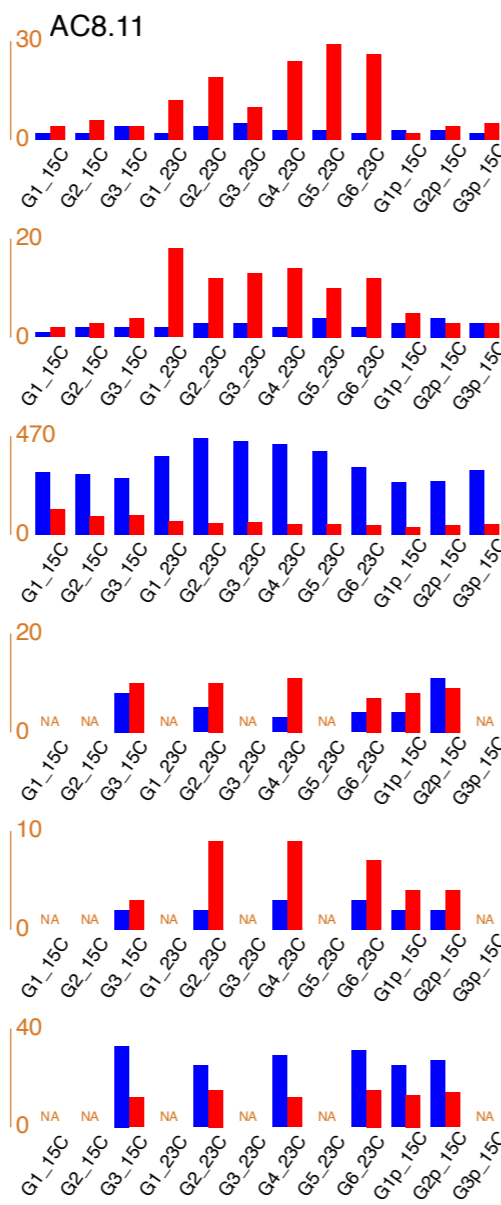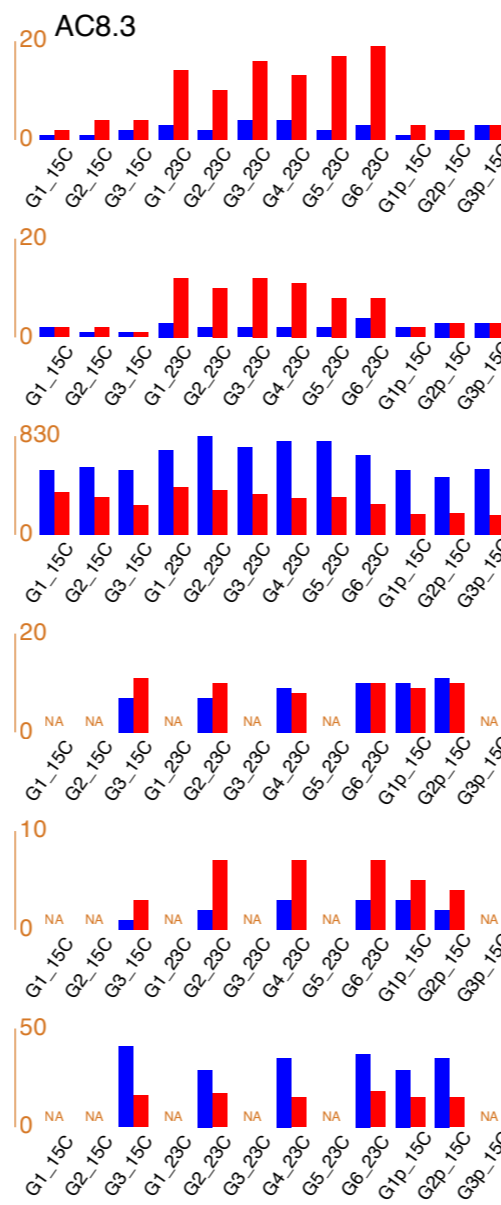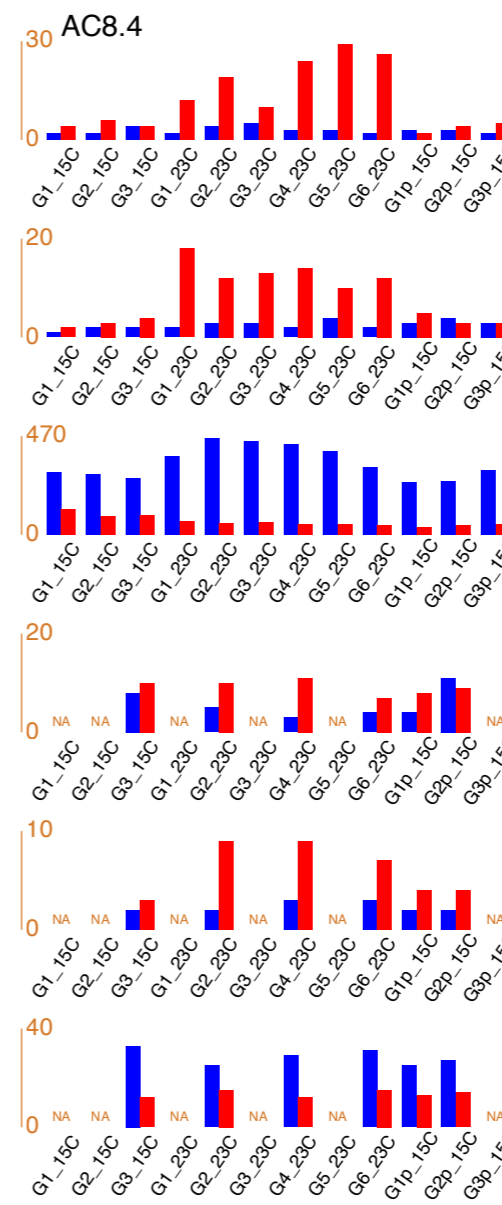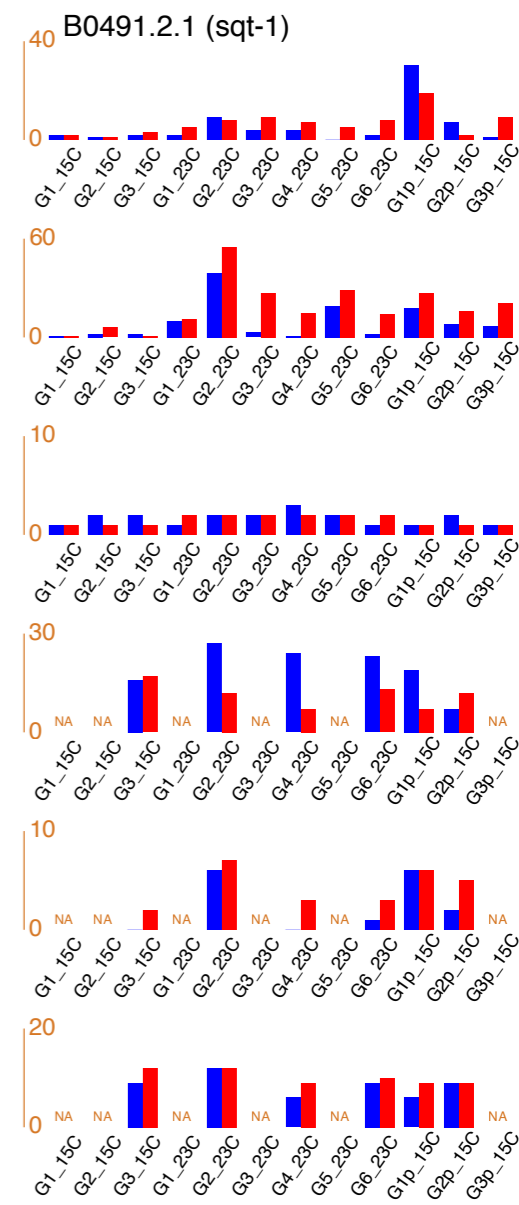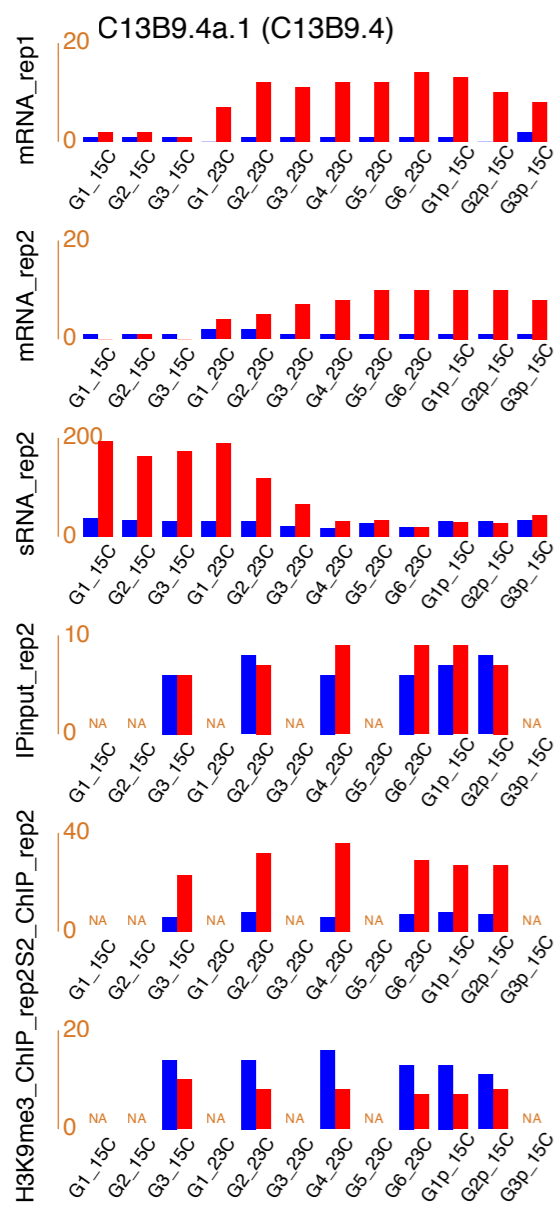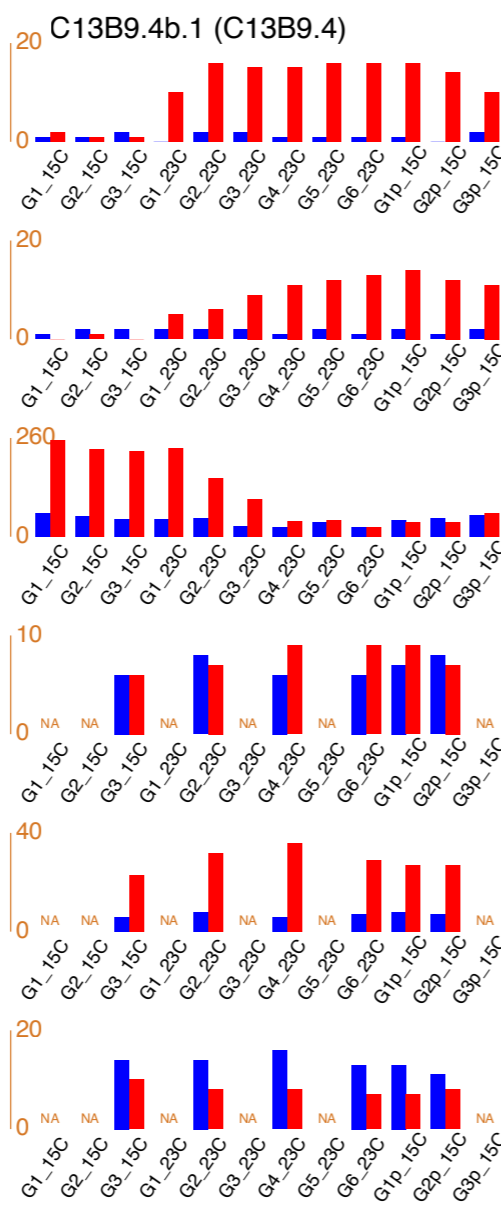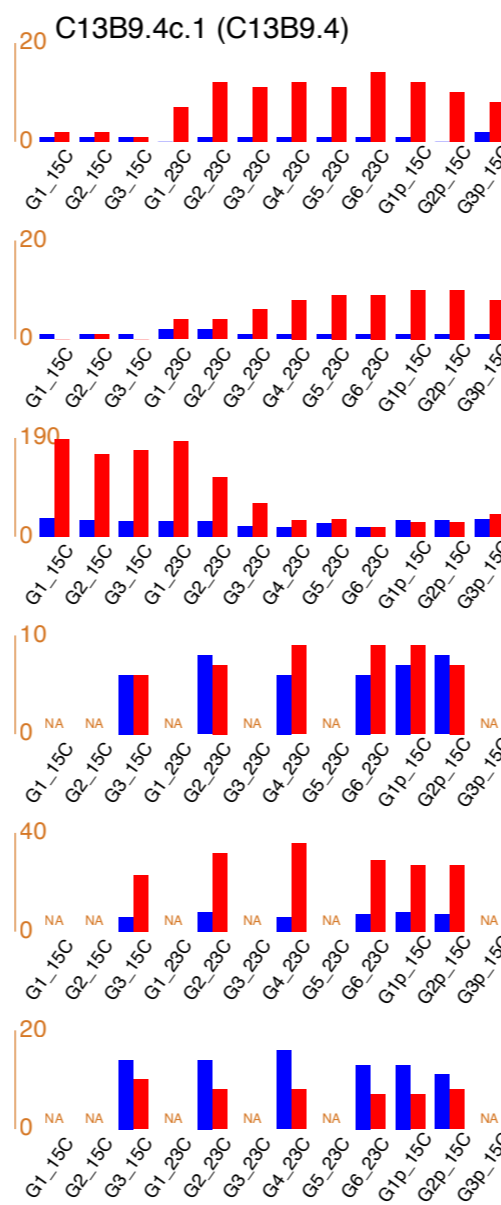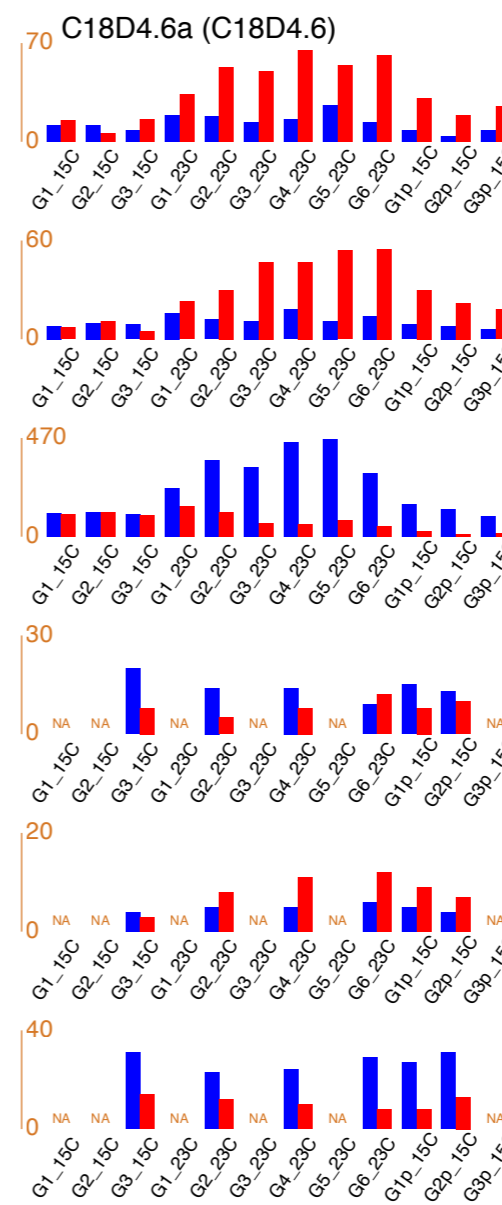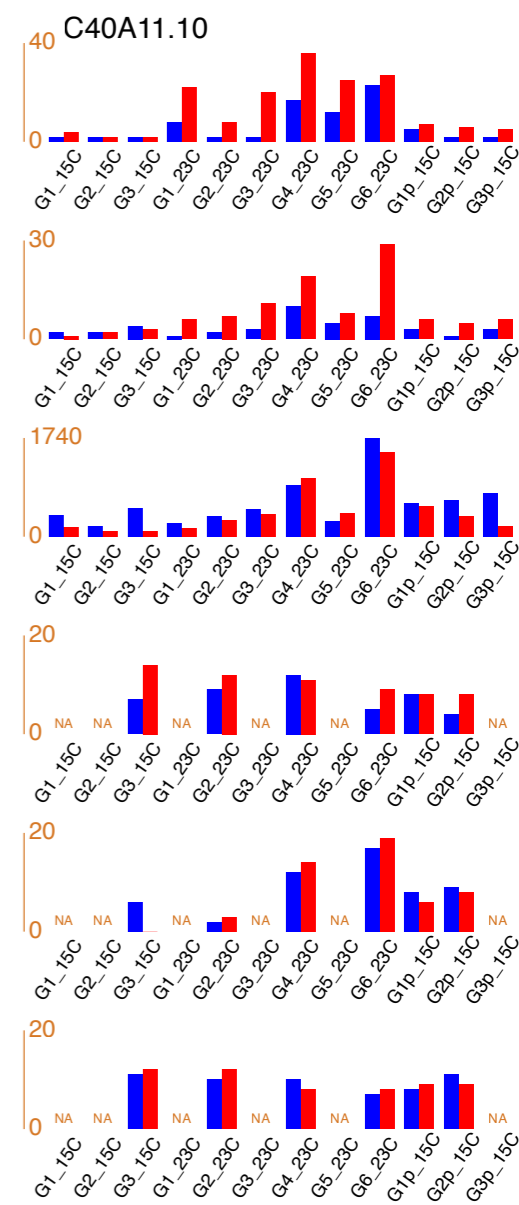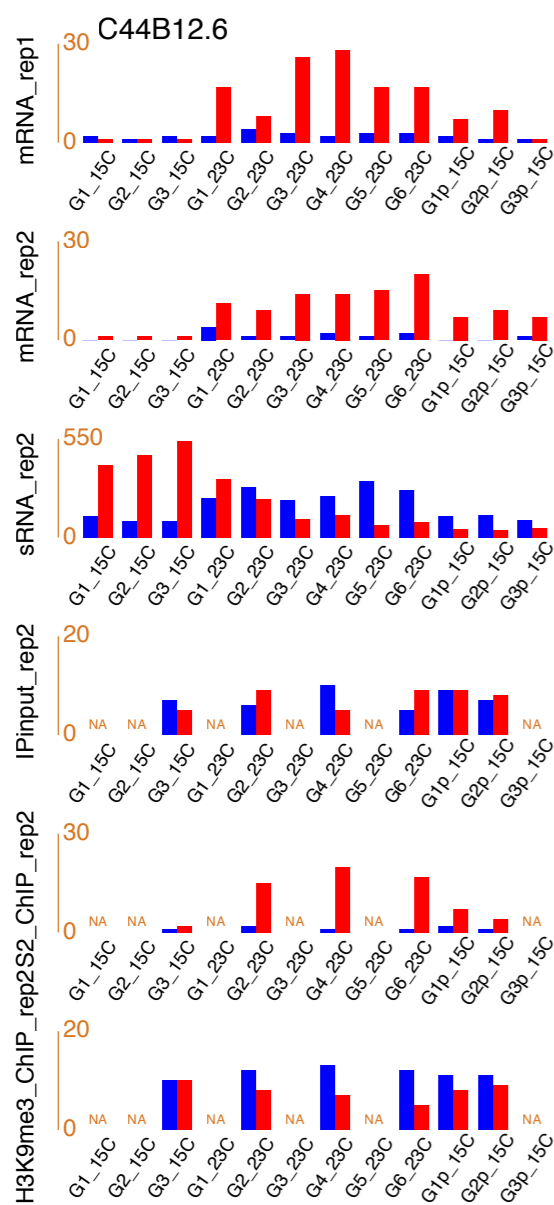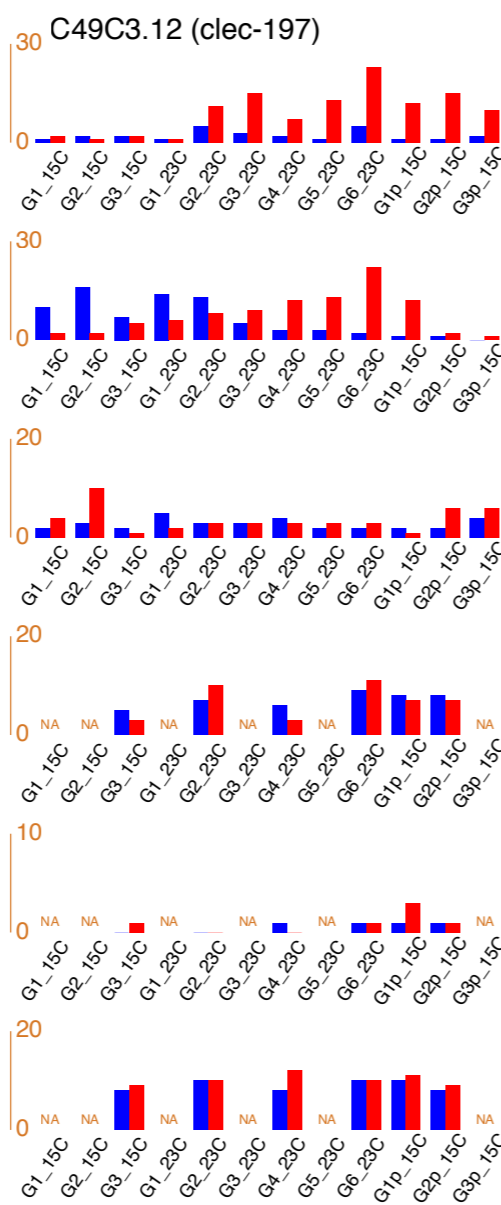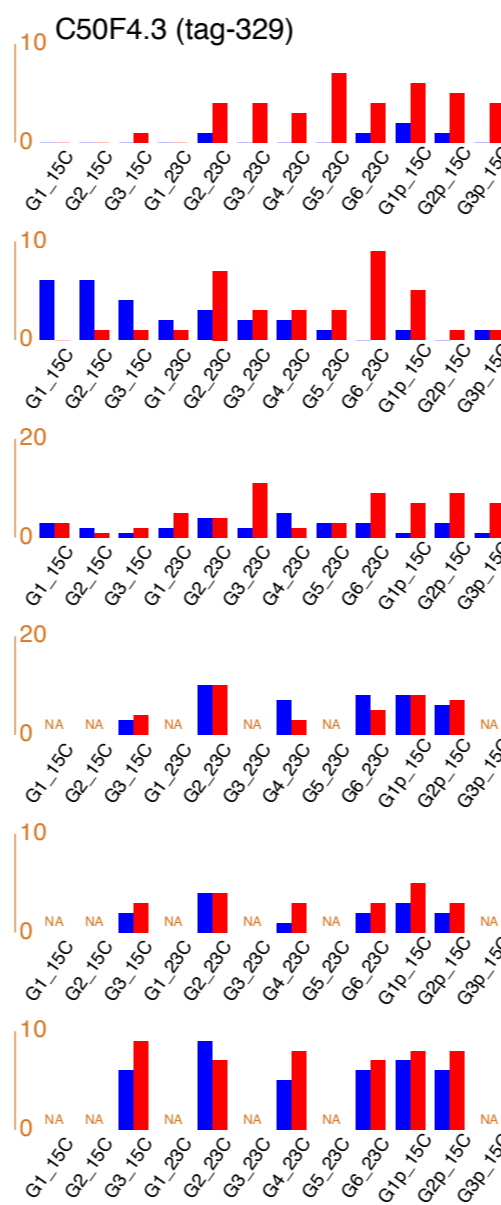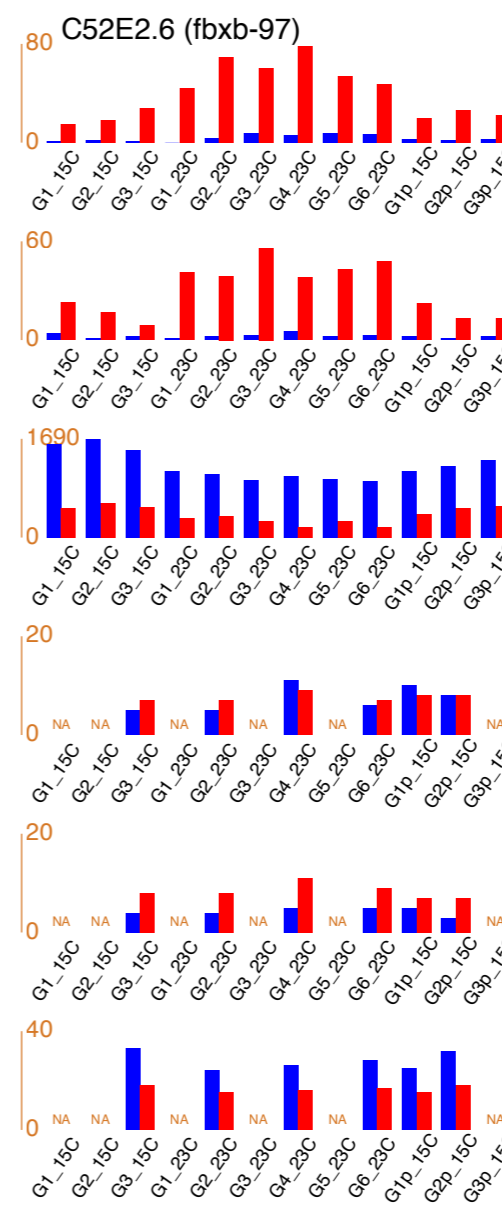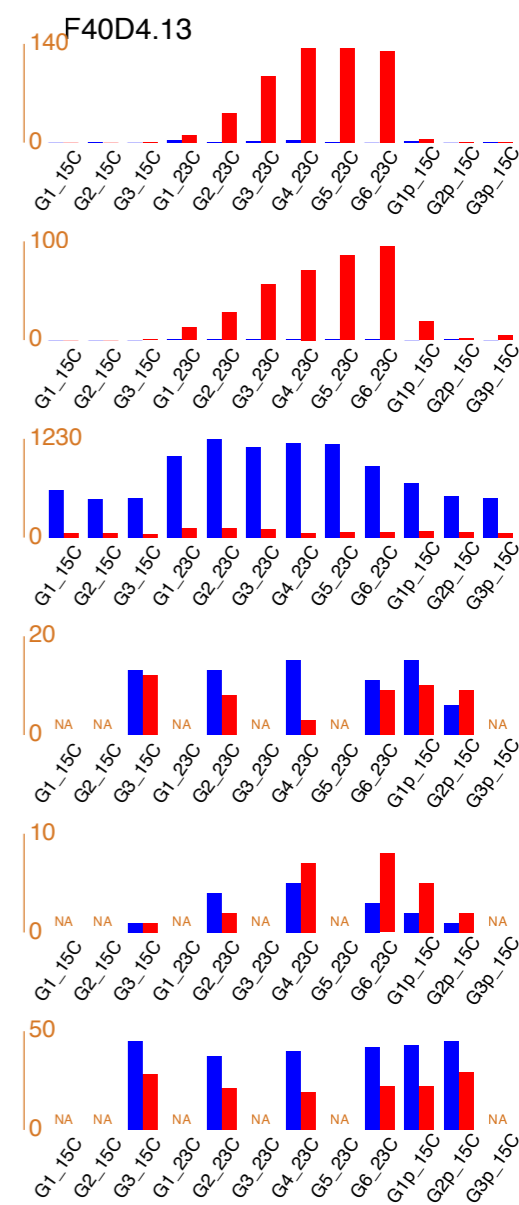

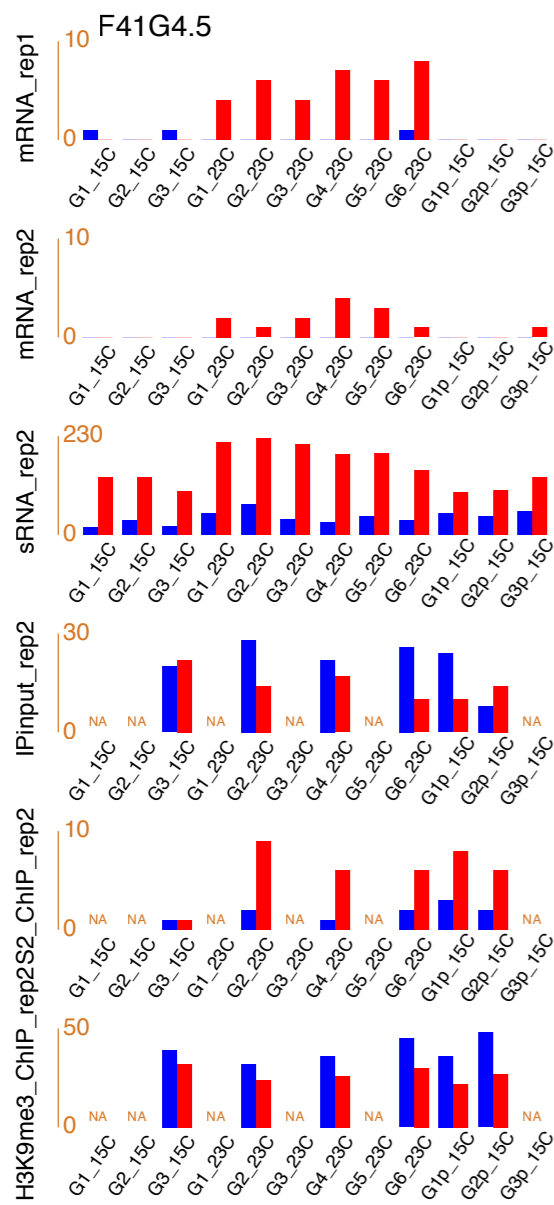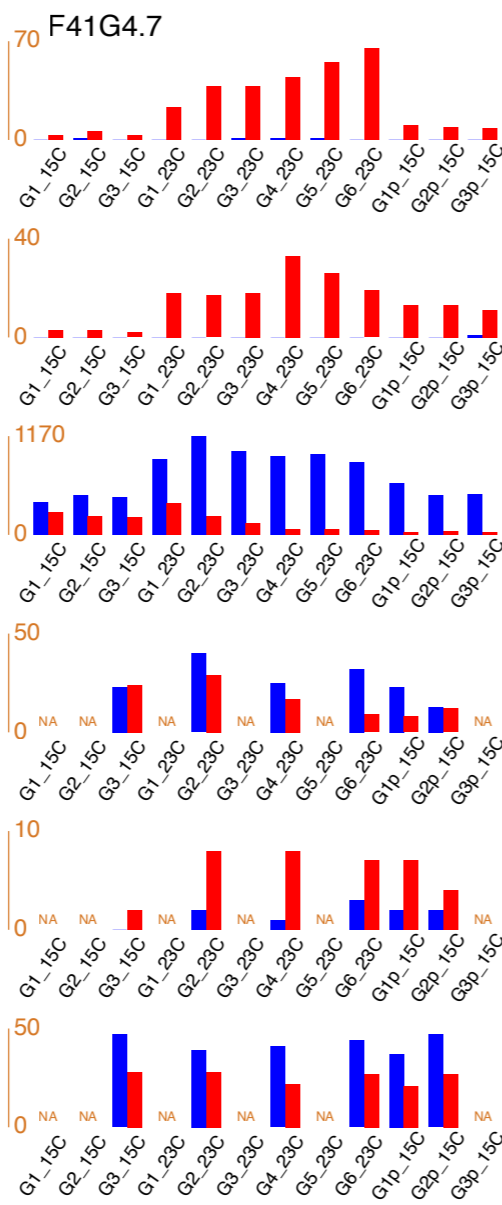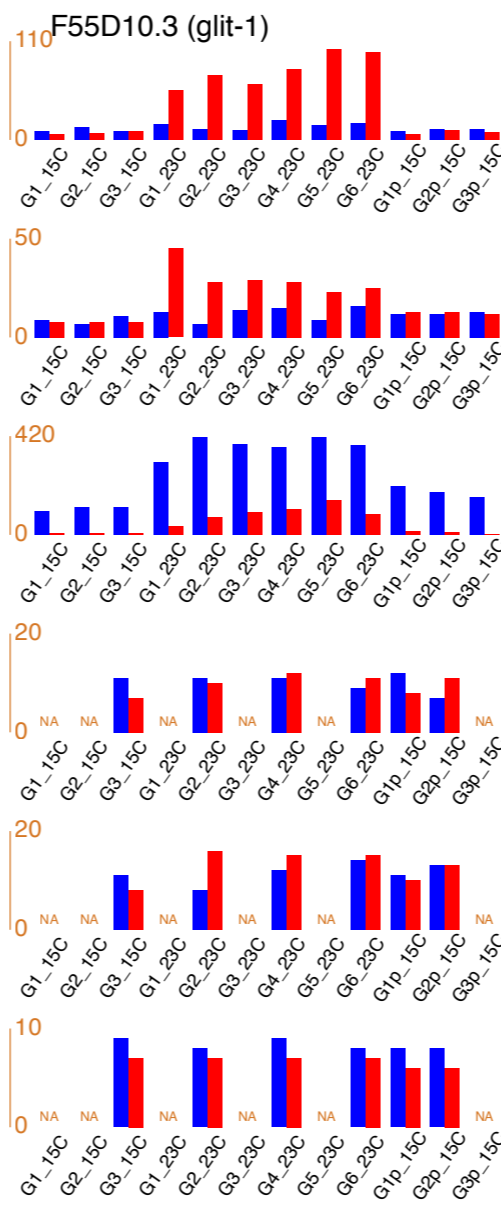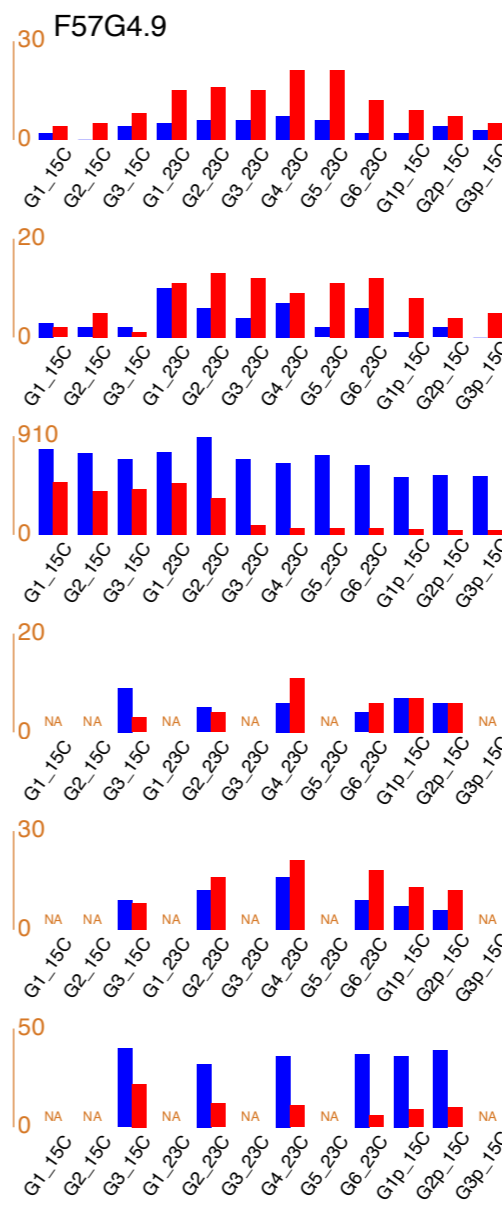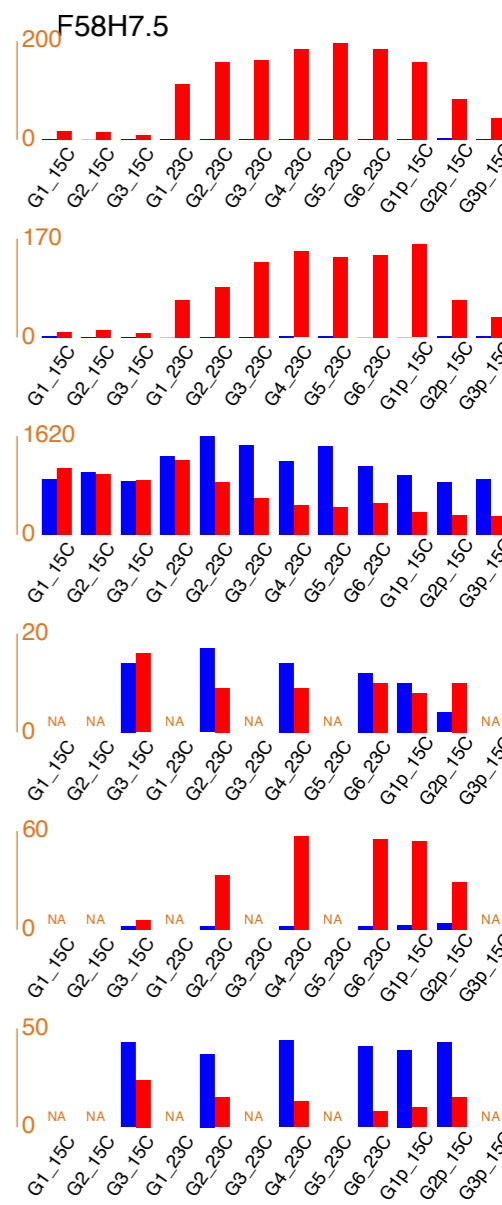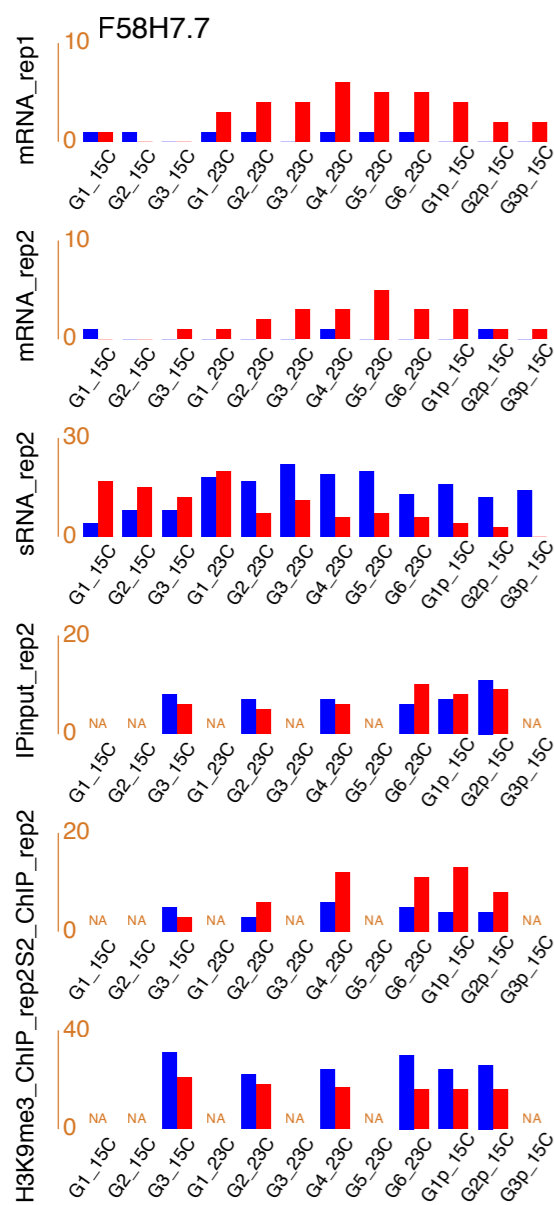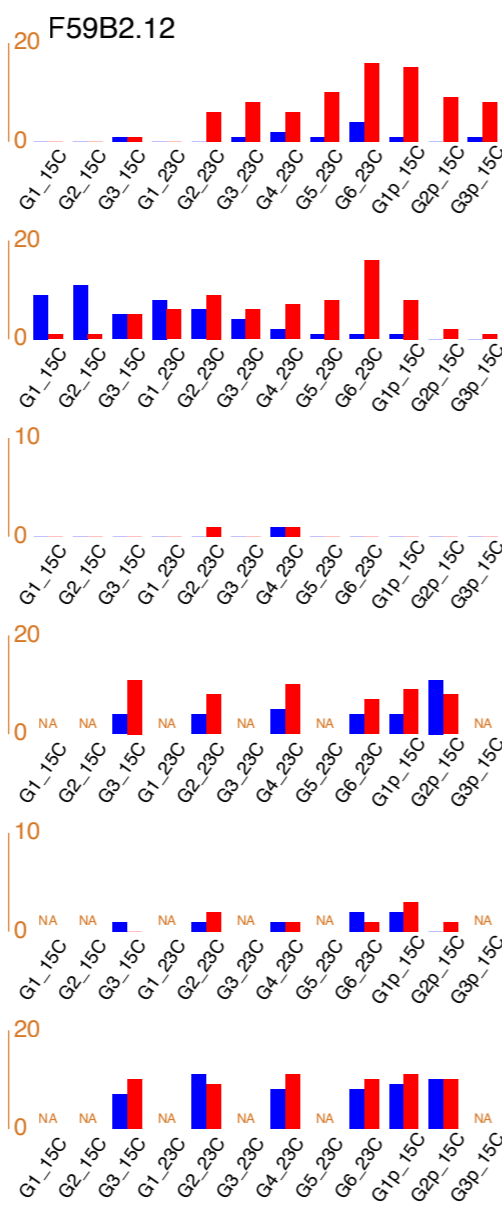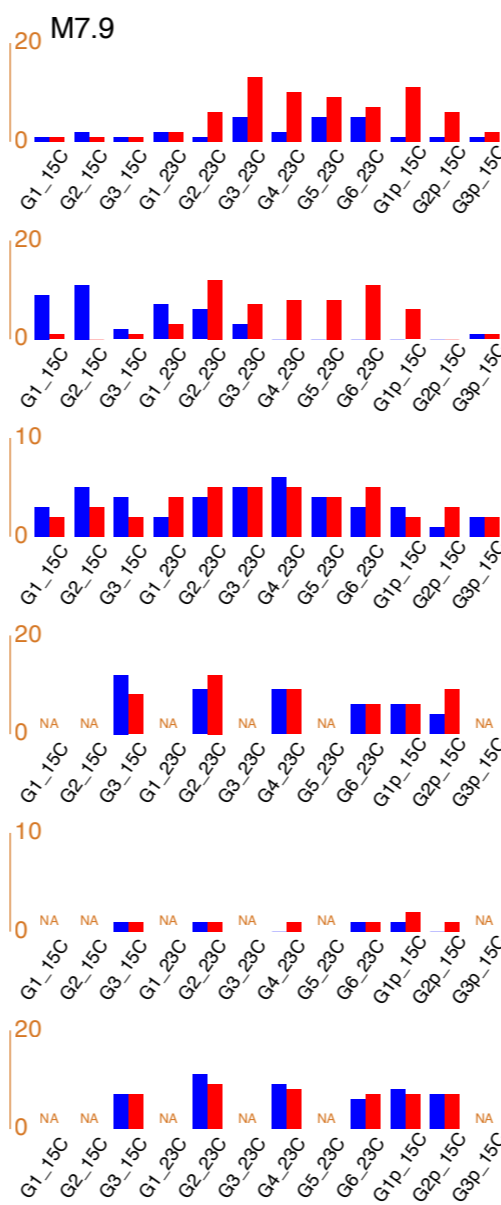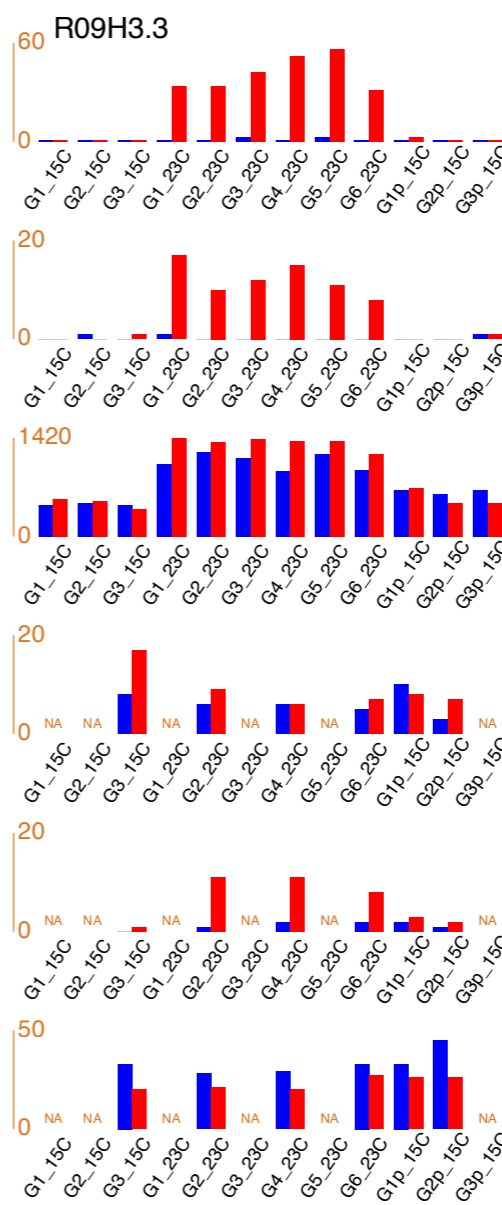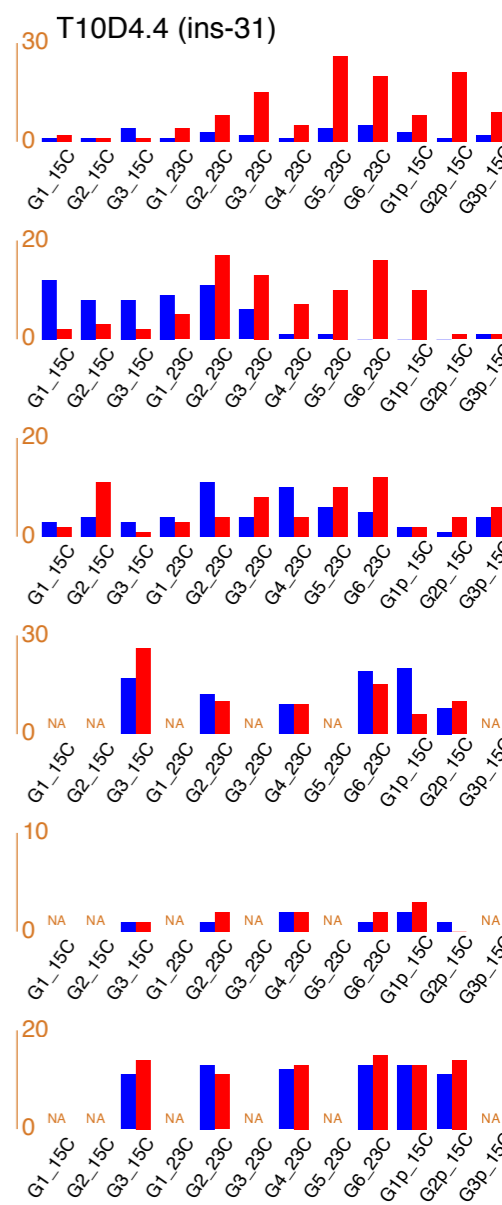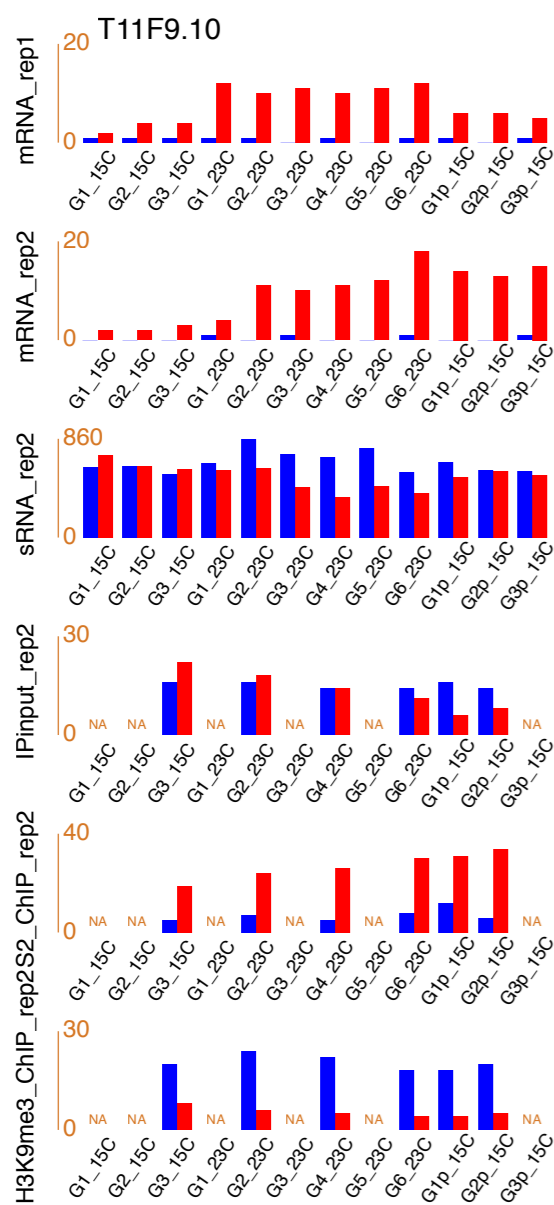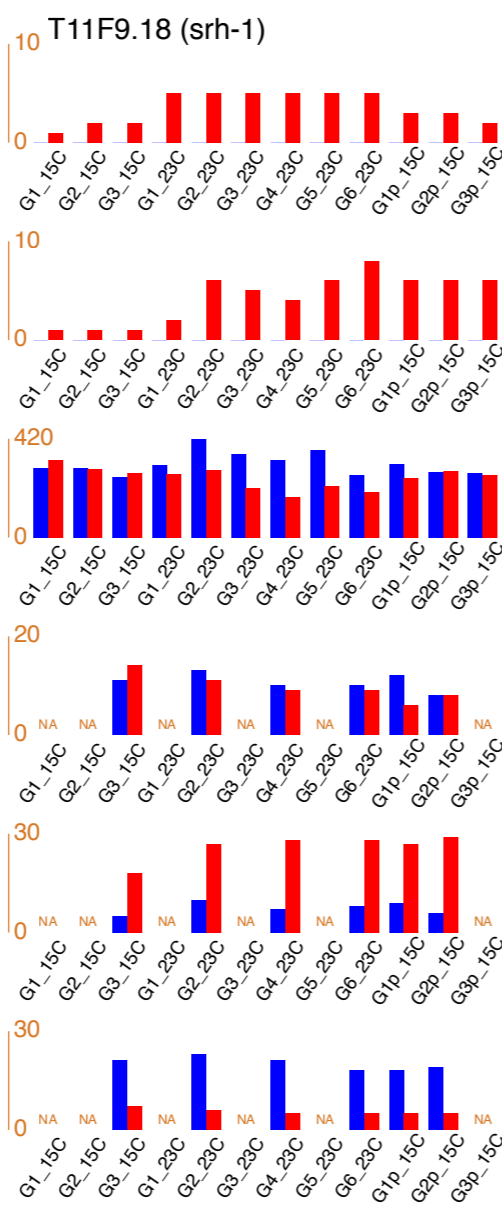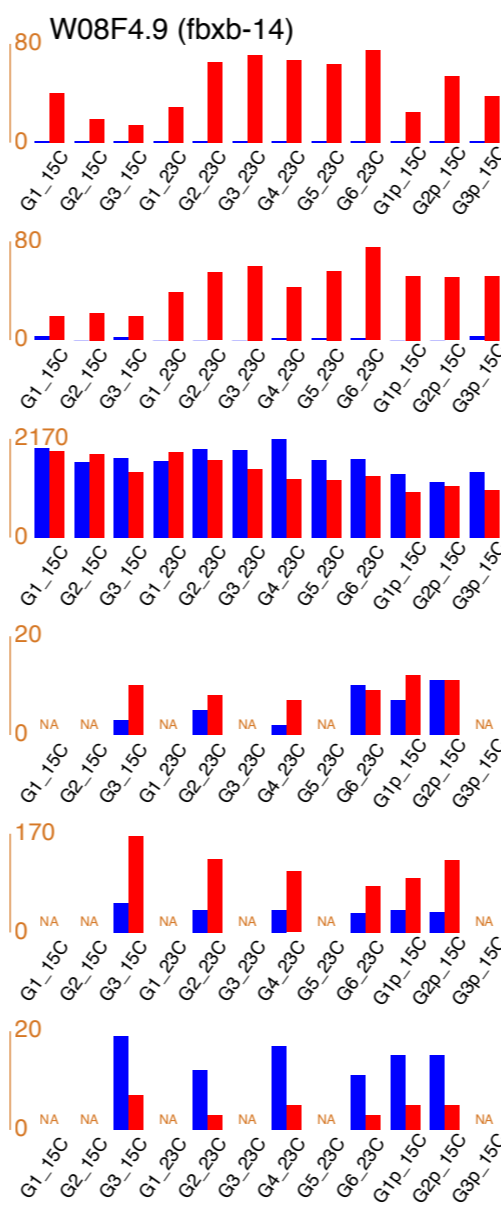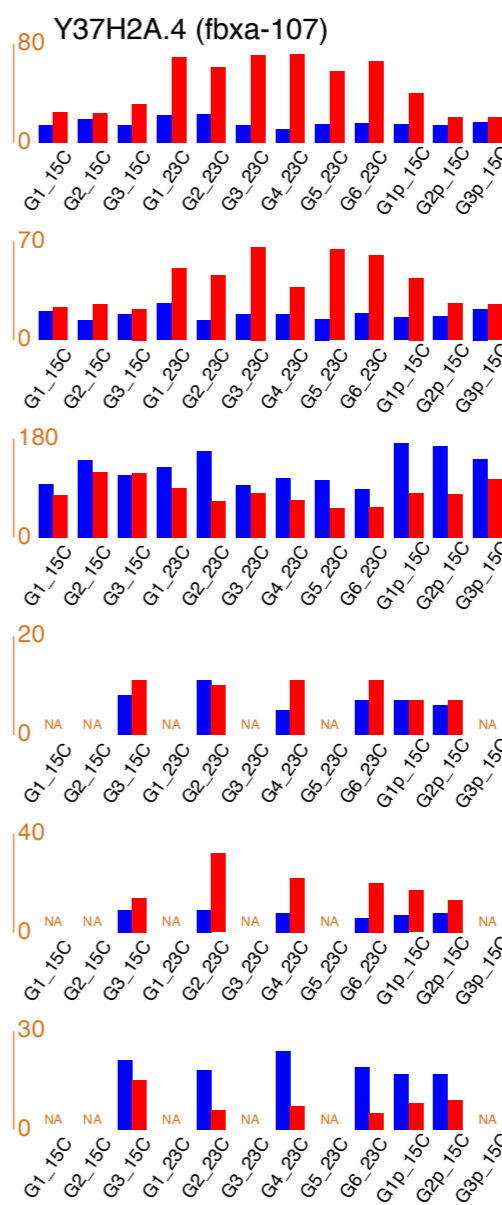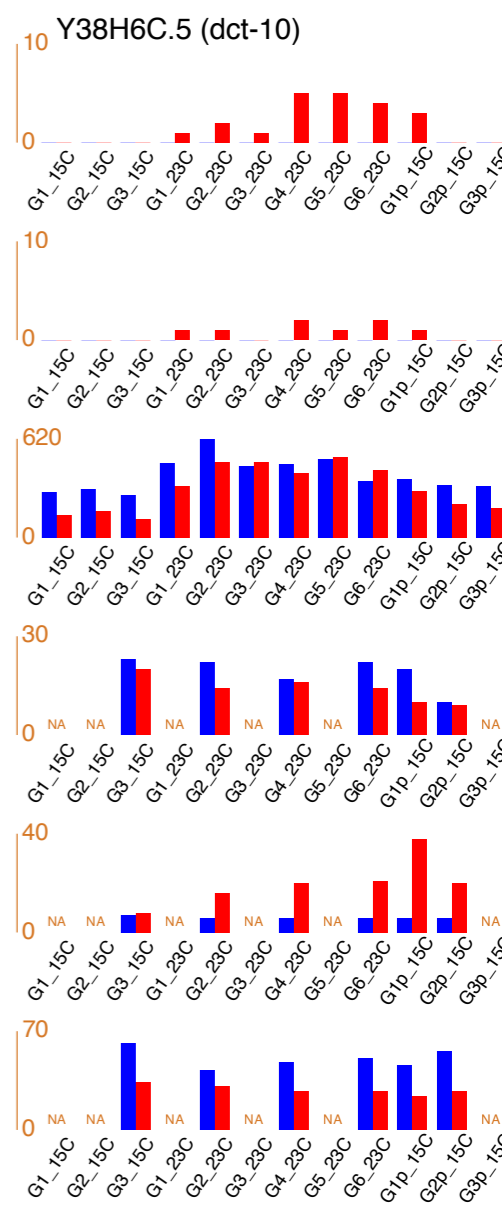

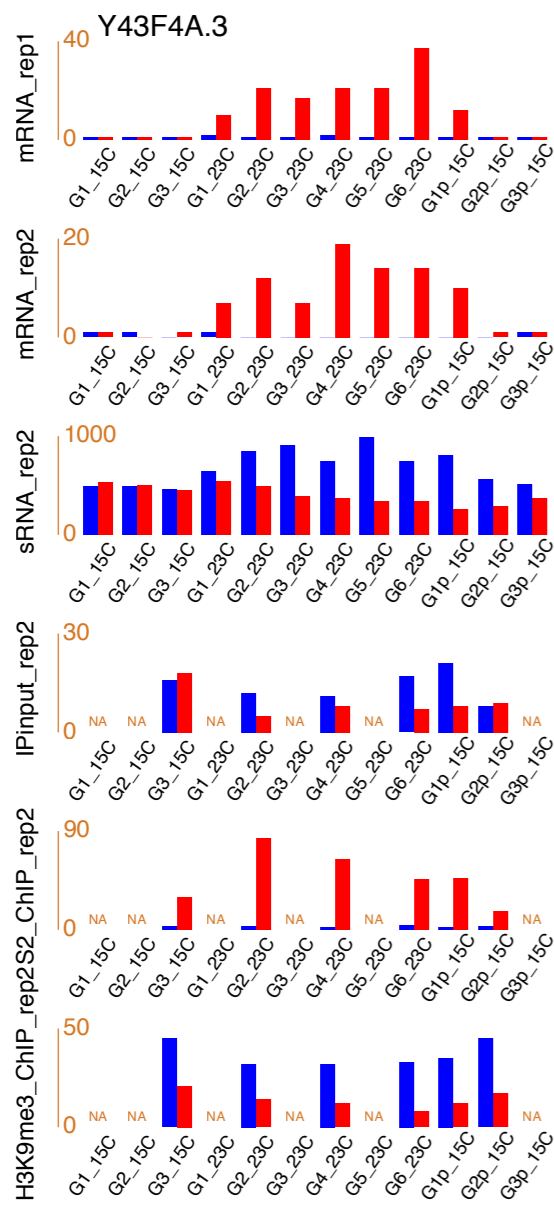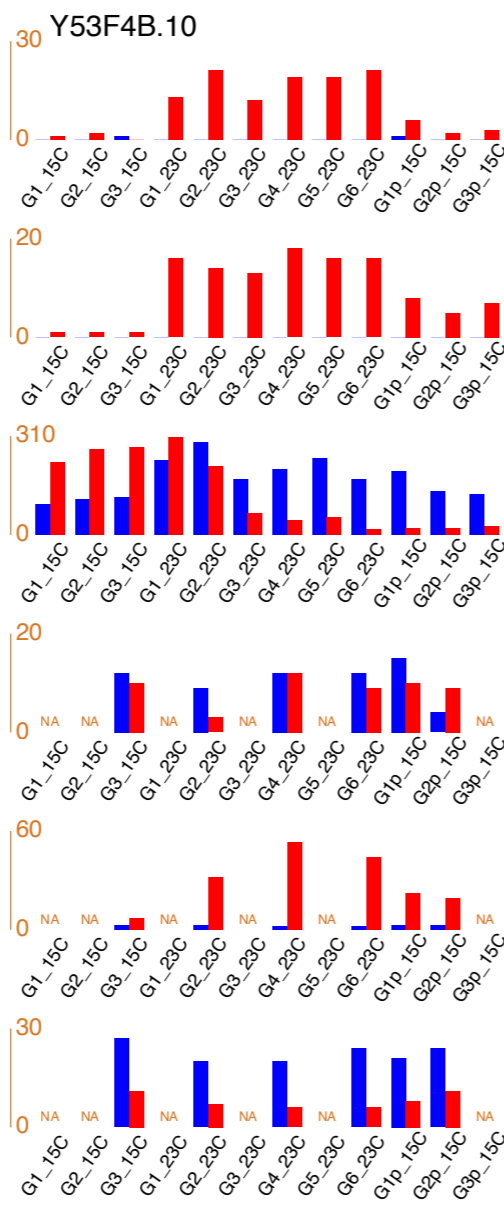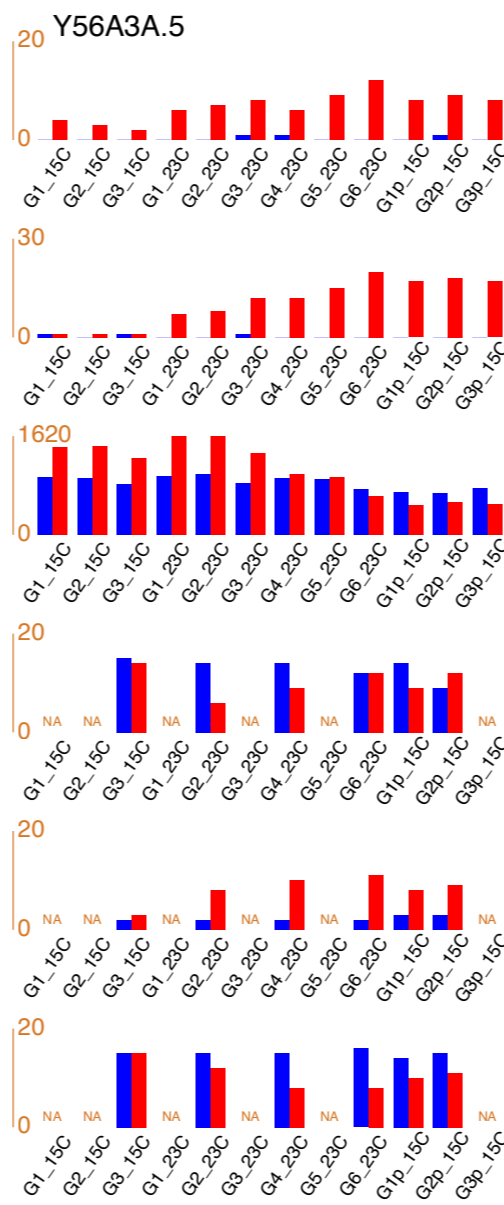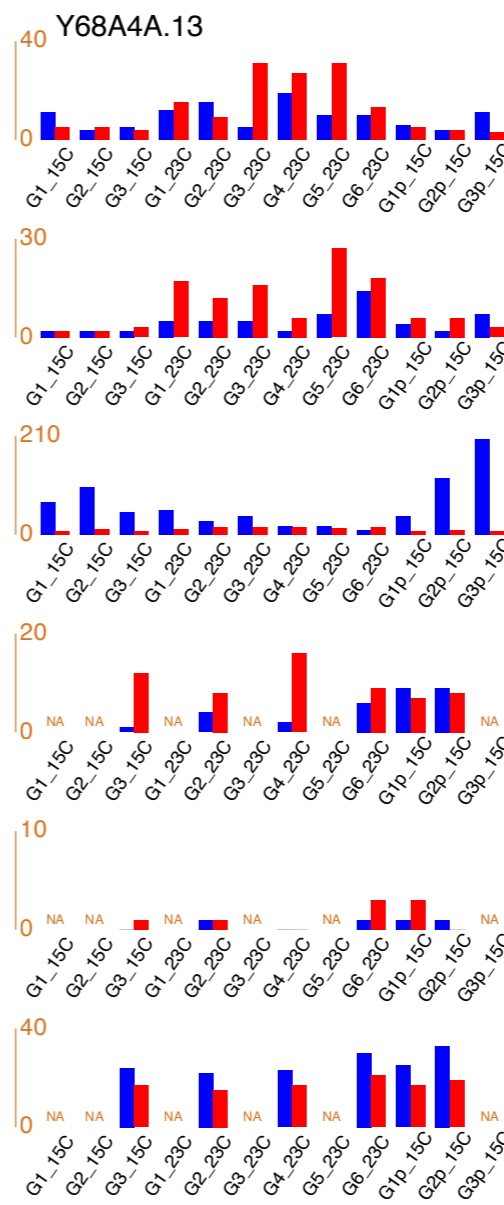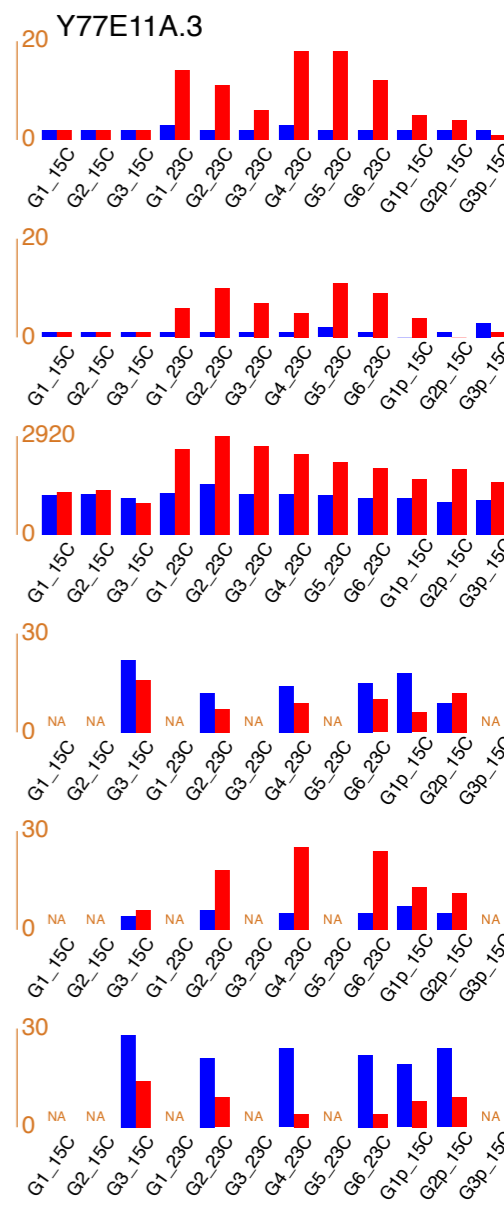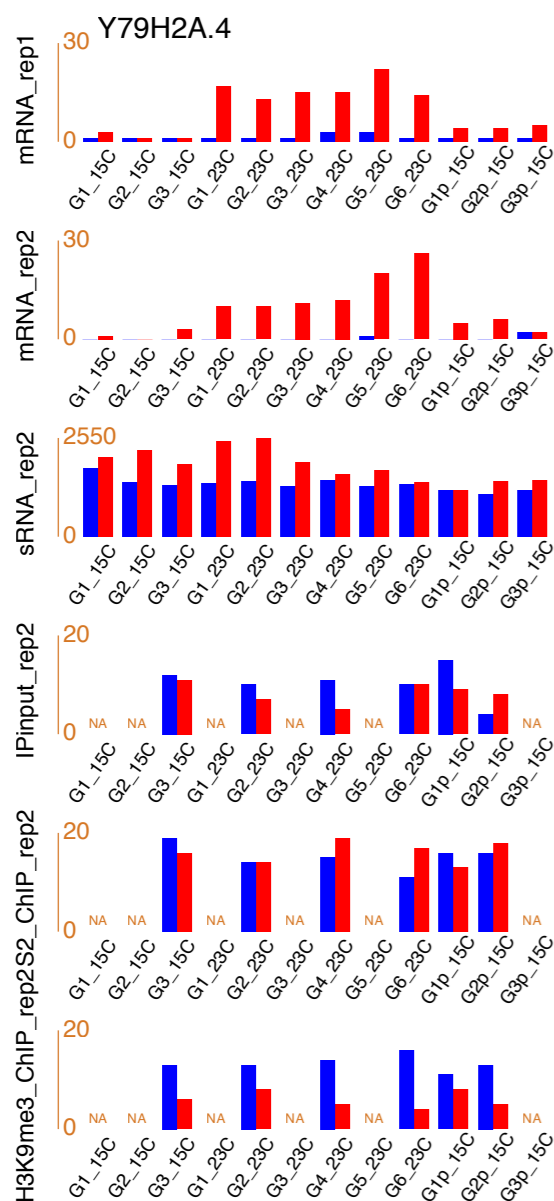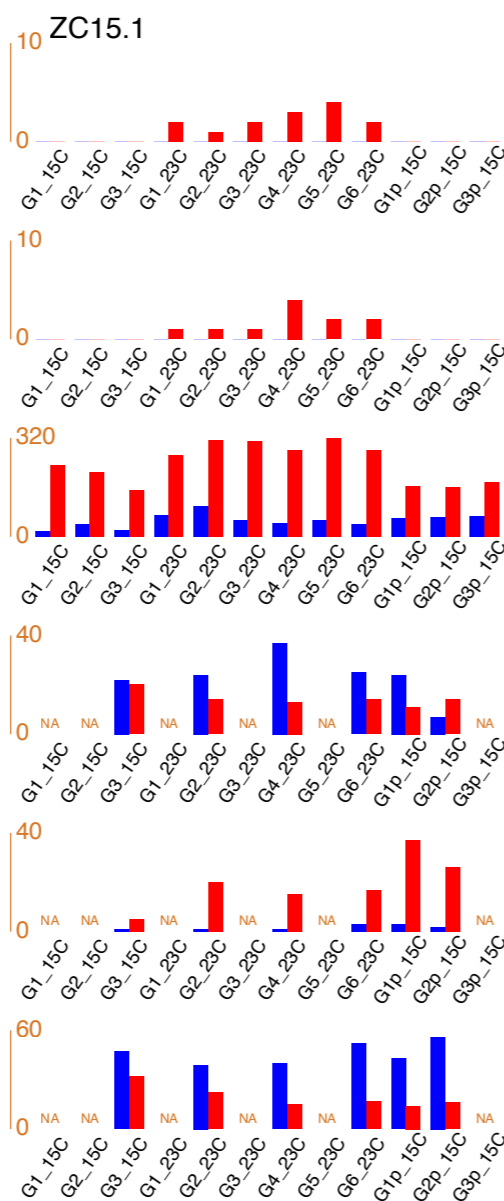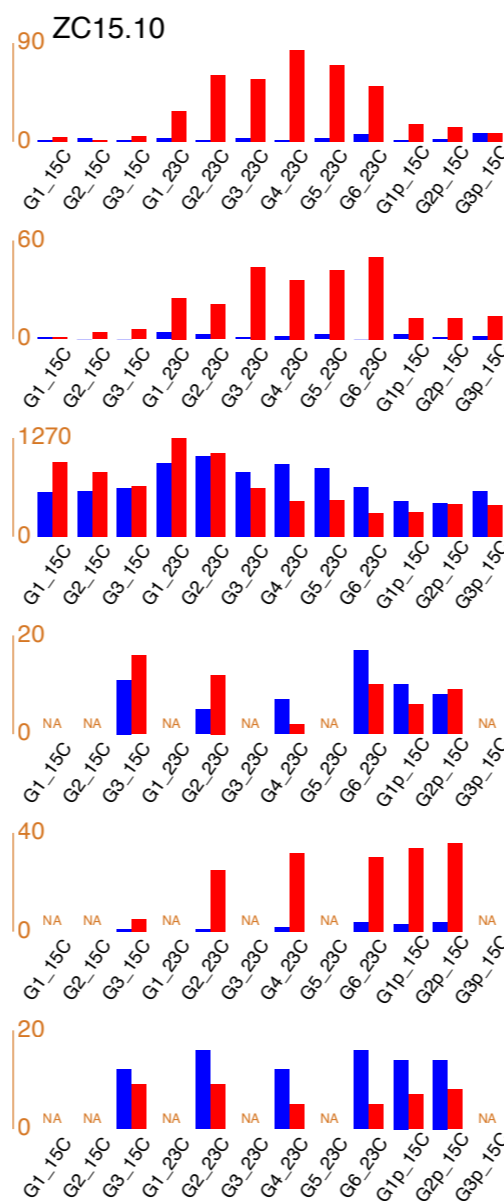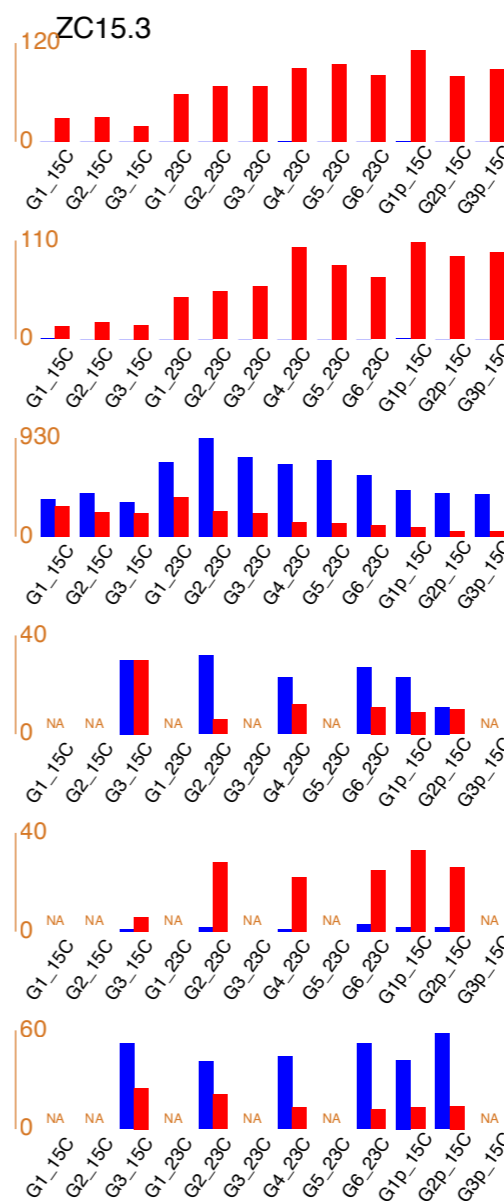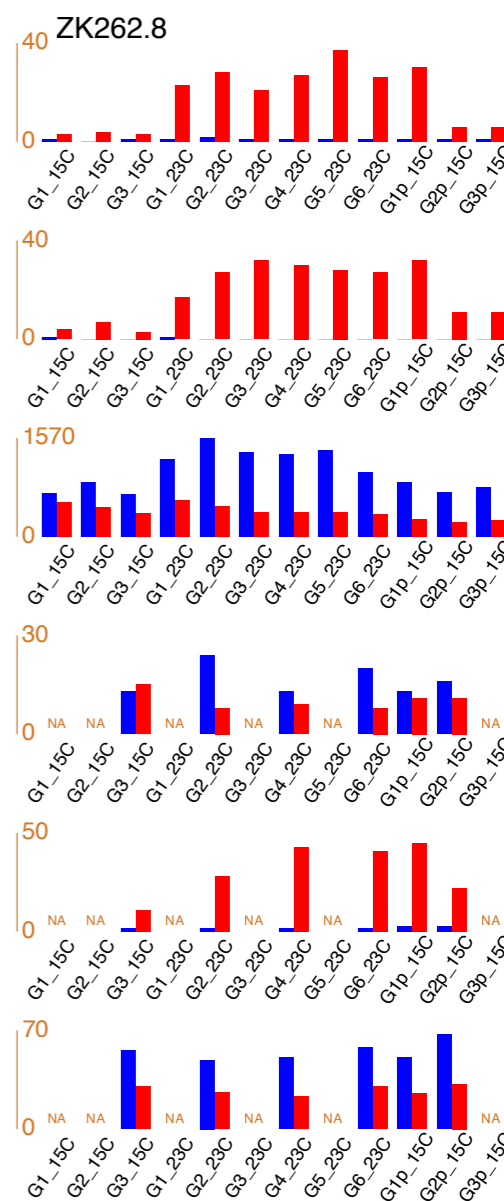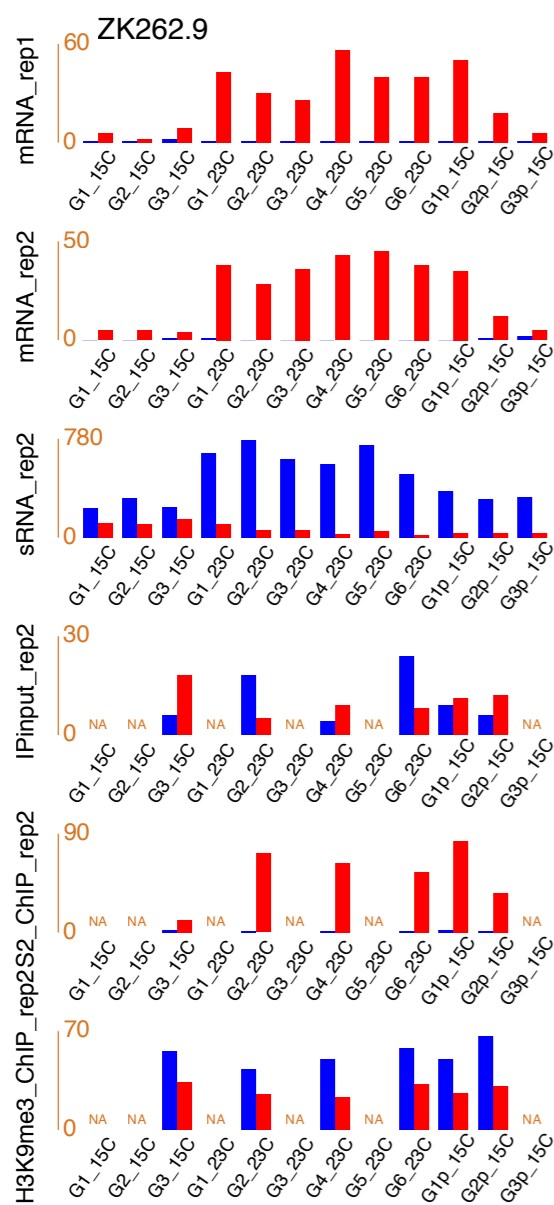

Supplement: Supplementary file 6 — 10.1186/s13072-016-0052-x Multigenerational mRNA, siRNA, Pol II, and H3K9me3 levels for the 41 high-stringent NHGs. [file 13072_2016_52_MOESM6_ESM.pdf]

a

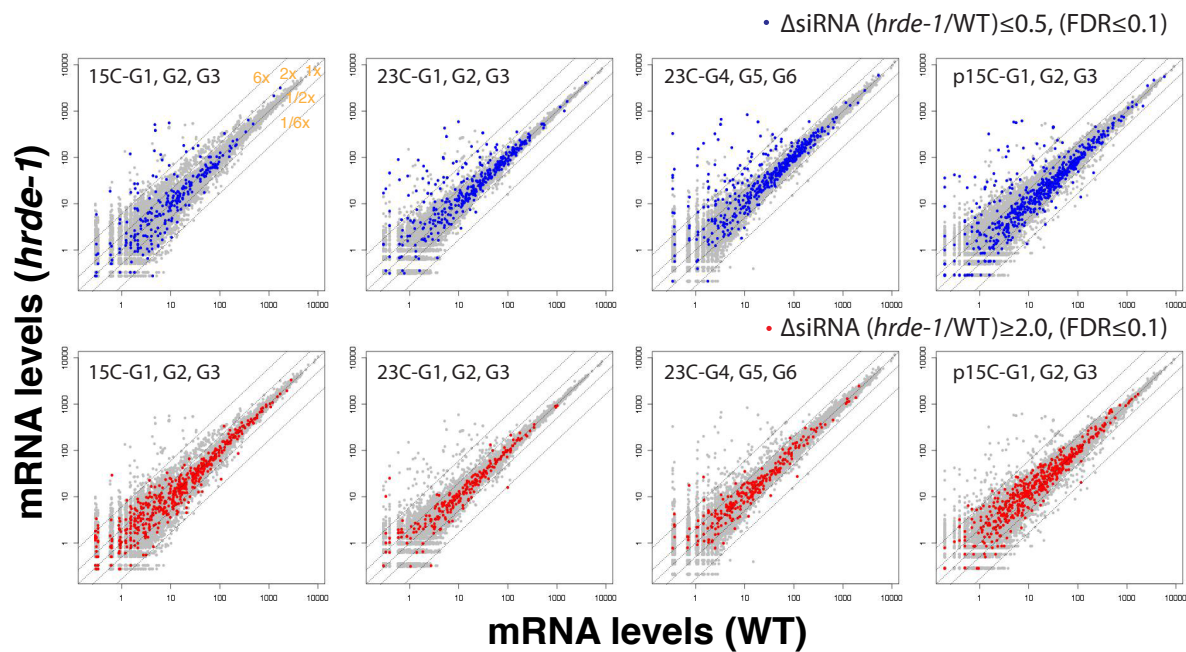

b

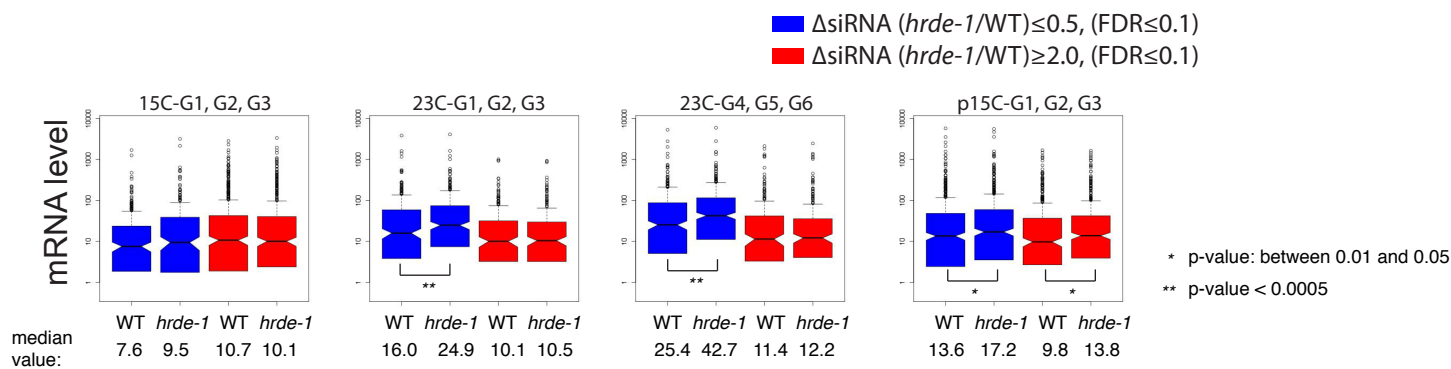

c

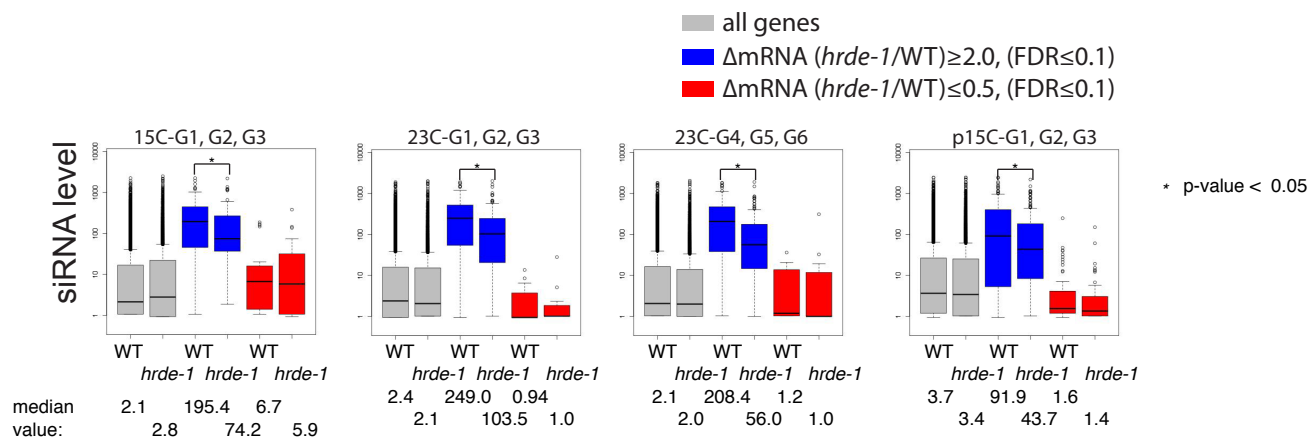

d

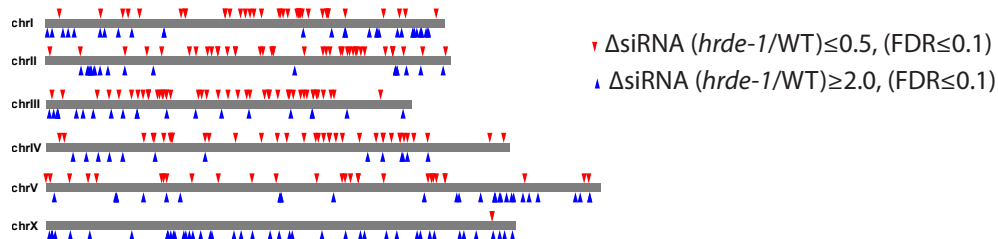

Supplement: Supplementary file 7 — 10.1186/s13072-016-0052-x Relationship between hrde-1-dependent siRNAs changes and hrde-1-dependent mRNA changes. (a) Scatter plots comparing mRNA levels of WT and hrde-1 mutant for genes with hrde-1-dependent siRNA changes highlighted for all four phases. (b) Box plot comparing mRNA expression levels between WT and hrde-1 mutant for genes with hrde-1-dependent siRNA changes for all phases. (c) Box plot comparing siRNA expression between WT and hrde-1 mutant for all genes and genes with hrde-1-dependent mRNA changes. (The results for phase III [23C-G4, G5, and G6] in panels a, b, and c were also shown in Fig. 5c, 5d, and 5e.) (d) Genomic distributions of genes with hrde-1-dependent siRNA changes in all four phases. Wilcoxon rank-sum test was used to calculate the p-values. [file 13072_2016_52_MOESM7_ESM.pdf]

a

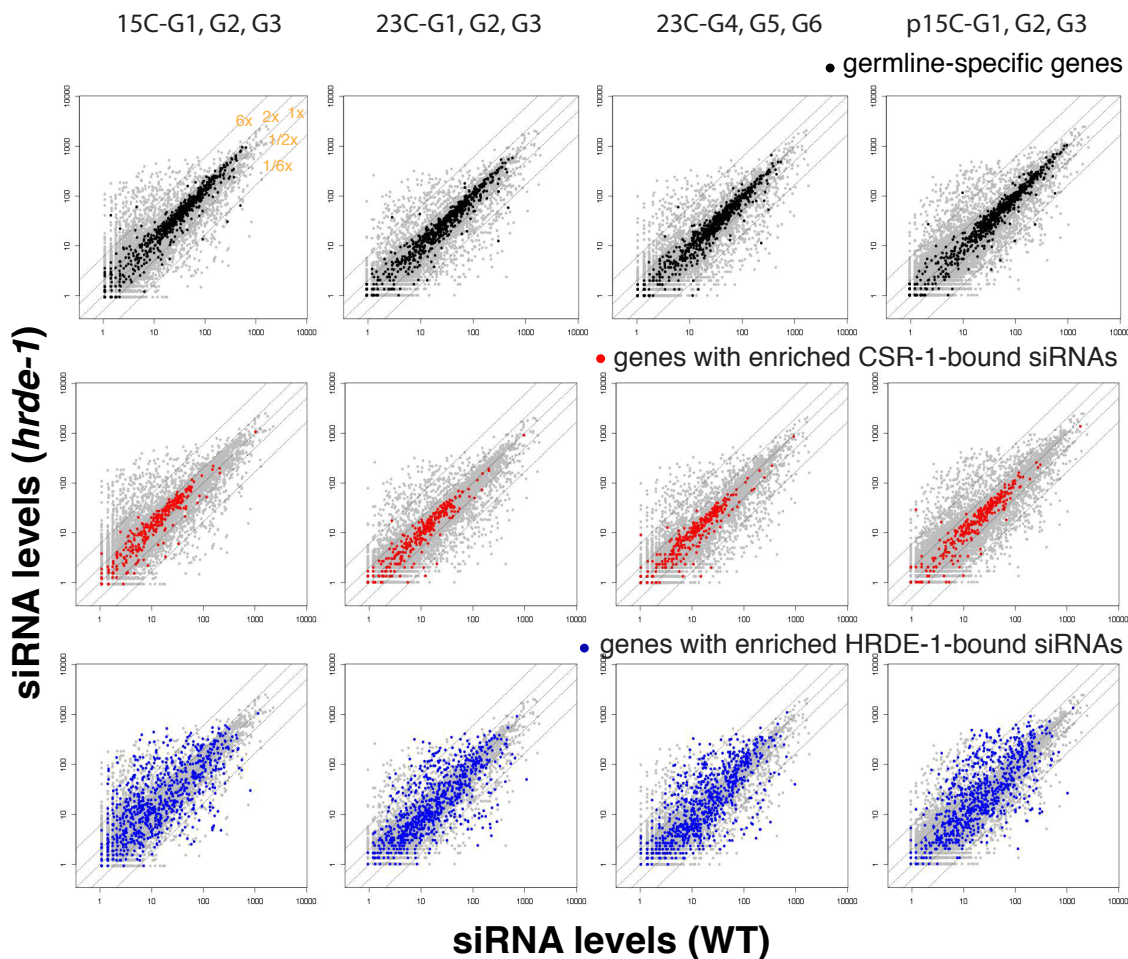

b

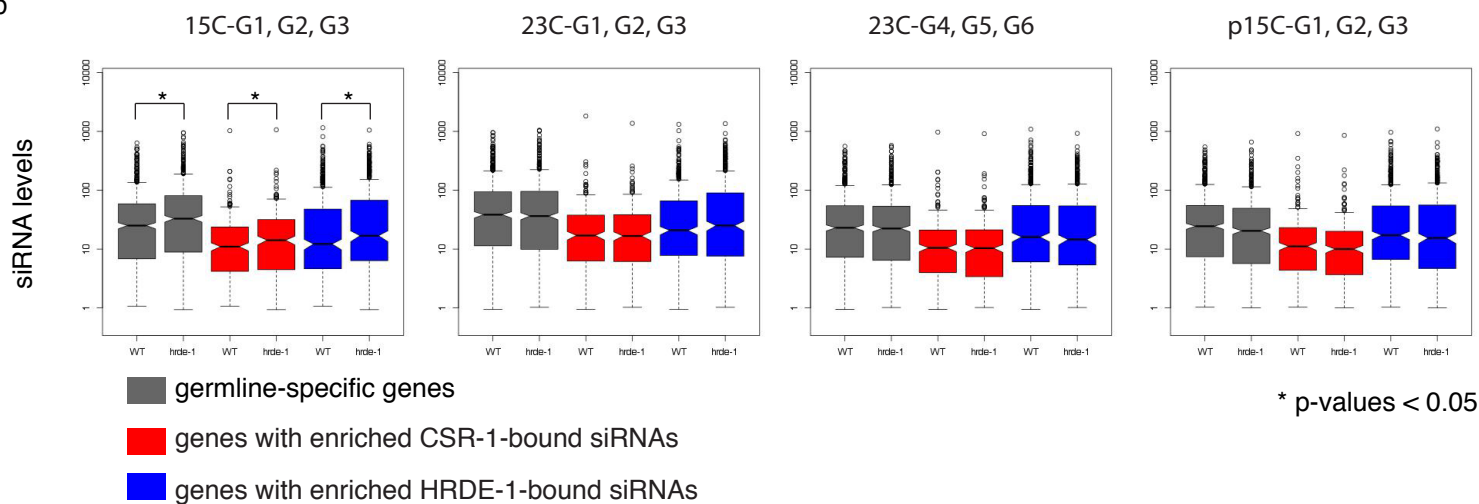

Supplement: Supplementary file 8 — 10.1186/s13072-016-0052-x (a) Scatter plots comparing siRNA levels of WT and hrde-1 mutant with germline-specific genes, CSR-1 targets, or HRDE-1 targets highlighted. (b) Box plots comparing siRNA levels of WT and hrde-1 mutant for germline-specific genes, CSR-1 targets, or HRDE-1 targets. Germline specific genes were defined by [24]. CSR-1 or HRDE-1 target genes were defined by using the published CSR-1-coIP [25] or HRDE-1-coIP siRNA profiles [10], respectively. CSR-1 targets: ∆siRNA (CSR-1-coIP/HRDE-1-coIP) > 10 and CSR-1-coIP siRNA (rpkm) > 40. HRDE-1 targets: ∆siRNA (HRDE-1-coIP/CSR-1-coIP) > 10 and HRDE-1-coIP siRNA (rpkm) > 40. [file 13072_2016_52_MOESM8_ESM.pdf]

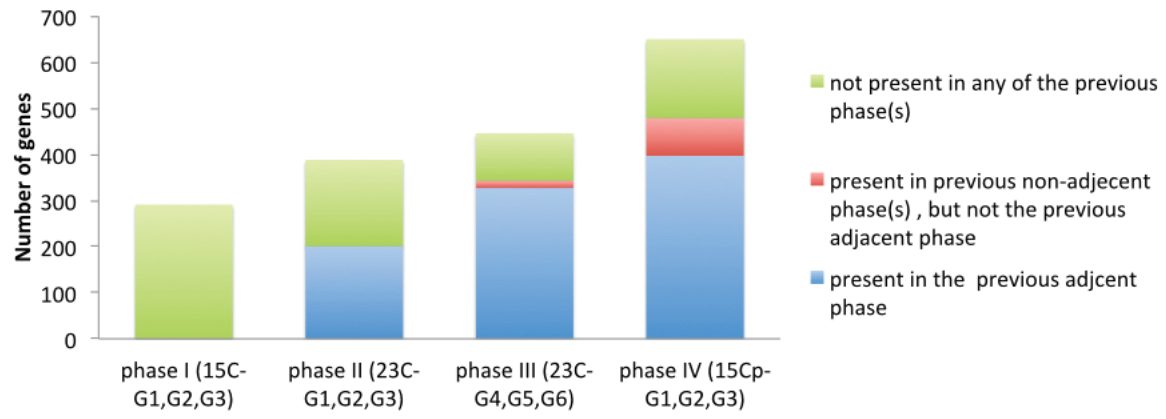

Supplement: Supplementary file 9 — 10.1186/s13072-016-0052-x Stacked bar graph showing the accumulation of genes with siRNA reductions in the hrde-1 mutant. [file 13072_2016_52_MOESM9_ESM.pdf]
